# Automated, non-invasive *Varroa* mite detection by vibrational measurements of gait combined with machine learning

**DOI:** 10.1038/s41598-023-36810-0

**Published:** 2023-06-23

**Authors:** Harriet Hall, Martin Bencsik, Michael Newton

**Affiliations:** School of Science and Technology, Nottingham Trent University, Clifton Lane, Nottingham, NG11 8NS UK

**Keywords:** Biophysics, Animal behaviour, Machine learning

## Abstract

Little is known about mite gait, but it has been suggested that there could be greater variation in locomotory styles for arachnids than insects. The *Varroa destructor* mite is a devastating ectoparasite of the honeybee. We aim to automatically detect *Varroa*-specific signals in long-term vibrational recordings of honeybee hives and additionally provide the first quantification and characterisation of *Varroa* gait through the analysis of its unique vibrational trace. These vibrations are used as part of a novel approach to achieve remote, non-invasive *Varroa* monitoring in honeybee colonies, requiring discrimination between mite and honeybee signals. We measure the vibrations occurring in samples of freshly collected capped brood-comb, and through combined critical listening and video recordings we build a training database for discrimination and classification purposes. In searching for a suitable vibrational feature, we demonstrate the outstanding value of two-dimensional-Fourier-transforms in invertebrate vibration analysis. Discrimination was less reliable when testing datasets comprising of *Varroa* within capped brood-cells, where *Varroa* induced signals are weaker than those produced on the cell surface. We here advance knowledge of *Varroa* vibration and locomotion, whilst expanding upon the remote detection strategies available for its control.

## Introduction

### Current knowledge on Varroa gait style

The *Varroa destructor* mite is an extensively researched species that specifically parasitises honeybees^1^. Despite the abundance of *Varroa* studies, there are few descriptions of their locomotory behaviour^2^. Of the gait characterisations that have been made, the focus has been on the forelegs, which are reported to function in the same way as insect antennae, detecting volatiles and mediating mite orientation towards honeybee hosts and conspecifics for reproduction^3–6^. *Varroa* are described as lifting their forelegs in an exploratory manner, leaving the 2nd, 3rd, and 4th pairs of legs to function as walking appendages^7,8^. Yet, these are merely anecdotal comments, and features relating to *Varroa* mite ambulation have not been quantitated or explored in any further detail.

#### Other mite and tick gait

A similar situation is seen regarding other mite and tick species. For ticks, studies only refer to locomotory activity, distance, and directional responses to hosts^9–12^. Many mite species are known to have modified forelegs that are used for a variety of purposes other than locomotion, and in the order Mesostigmata, to which *Varroa* belongs, the function is (like *Varroa*) sensory in nature^13^. However, gait specific features are not well-researched in mites, and locomotion is more commonly discussed in relation to speed, grasping, or jumping ability^14,15^, with limited observational references to six-legged gait^16–19^.

#### Six-legged arachnid gait

As *Varroa* walk with their forelegs raised above ground, using only the other six for ambulation, it is perhaps likely that their locomotory style is akin to that of other arachnid species also known to move using only six out of eight of their legs. For example, the jumping spider *Myrmarachne formicaria* can mimic the motion of its ant prey by lifting its forelegs to imitate antennal behaviour^20^. There are other related orders within the Arachnid class that, like *Varroa*, have evolved legs that function as sensing systems, leading to tripodal gait. Harvestmen (Opiliones) have four different gait-types, and never use the second pair of legs, which instead are used for environmental probing^20,21^. Whip spiders (Amblypygi) and whip scorpions (Thelyphonida) have what are described as antenniform forelegs that have developed a sensory function and play no role in ambulation^14,22,23^.

#### A unique gait style for Varroa?

However, a detailed review on locomotion (including gait style, step cycles, running speed and morphological adaptations) in the arachnid class notes that gait types appear to vary far more in this group than insects^14^, and it is therefore possible that *Varroa* locomotory style is unique to that of other arachnids, mite or otherwise. The species has unusual anatomical features noted in its muscular structure, and its body plan varies substantially to that of other mites^2^. The coxae (first joint) of the legs are immovable in other arachnid species, yet in *Varroa*, retractor muscles in the coxae instead attach to an endosternite (part of the internal skeleton), rather than the body cuticle, enabling greater movement of the legs. Additionally, the somatic muscles crisscross the body, forming a knot, providing *Varroa* with another exceptional feature^2^. Investigation into the gait of this species, which is described as being both efficient and rapid^2^, is therefore likely to provide interesting further insight into *Varroa* biology. In this study, we make a start in this direction, and investigate *Varroa* gait in terms of its vibrational signature, primarily as a tool for its detection in honeybee colonies, but also to provide novel insight into the unique pattern of *Varroa* walking style via its electronic features.

### Varroa mite infestation in honeybee colonies and the importance of its detection

*Varroa* act as a vector of disease and negatively influence a variety of bee functions and biological processes, including cognition and immune system activity^1,24^. If colonies are not well managed for mite infestation, they are likely to die between 6 months and 2 years after the parasite takes hold^25^. The importance of monitoring and managing this high impact species within honeybee colonies is therefore paramount.

Monitoring for mite populations is critical in order to allow the appropriate timing of control methods, as over treating with acaricides can (i) have adverse effects on honeybee biological processes and (ii) can also lead to *Varroa* resistance^26,27^. Not all colonies within an apiary will require treatment, i.e., those that lack, or have low levels of mites should be left untreated (but continue to be monitored) to lower beekeeping efforts and reduce over-treatment risks^28^. Current approaches for identifying *Varroa* presence and infestation levels in honeybee colonies are practical in nature, requiring regular apiary visits and often causing disruption to the colony through the removal of bee and brood samples^27,29^. Remote, non-invasive measurements of *Varroa* levels could therefore greatly benefit beekeeping and colony health for two reasons: (i) the beekeeper can be informed of the most suitable time to treat their hives through continuous measurements of mite population, and (ii) the method would require fewer in-person visitations/inspections, reducing colony disruption and freeing up more of the beekeepers’ time.

Remote mite monitoring is a new field of investigation that offers the potential to greatly improve colony management practices for the reasons discussed above. At present, olfactory, video, and acoustic measurements are being investigated for their success and reliability as non-invasive surveillance techniques, but so far, there are several limitations and disadvantages to these methods, preventing their implementation in commercial products.

Only one research group has so far compared the acoustic signature of a *Varroa* infested colony with that of a healthy colony, and the data used by the authors suffered from low quality data and low replication due to a small number of honeybee colonies^30^. This is disadvantageous, as the interpretation of different colony states is a critical part of data analysis when it comes to remote monitoring, so that beekeepers can be correctly informed of hive status^31^. High quality data coming from numerous colonies is essential for building algorithms that can discriminate one colony state/event from another^32^, otherwise there is the risk of coincidental numerical categorisation.

Video capture has also been implemented as a *Varroa* detection method but is flawed by (i) the large amount of data produced (typically 20 to 100 fold higher than audio data, for example), (ii) difficulties in visual mite identification as they can reside on adult bees in a variety of positions, and (iii) the simple fact that video is only currently useful at the hive entrance, the only location where there is enough light and space to suitably collect information visually^31,33,34^.

Gas sensors, which collect olfactory measurements based on the chemical composition of the hive atmosphere, are the latest method to be scrutinised^35^. Most gas sensors are unfortunately only sensitive to one type of simple compound, and as the composition of beehive air, and that of *Varroa* infested hives is known to be highly complex this currently means that many devices are required for one colony^35,36^. Other disadvantages include the need to be protected from propolisation, leading to the flaw that they are prone to errors if the measuring system is not cleaned regularly, which limits continuous monitoring possibilities^35,37^.

### Investigating Varroa gait and a novel approach to remote mite detection

We here present the first known quantitative characterisation of *Varroa* gait, based on its vibrational signature, and then use this as the basis of a novel, non-invasive mite detection approach in honeybee colonies. We use accelerometer sensors, which are small devices that measure vibration by outputting an electrical signal that is proportional to the acceleration it experiences^38^. This type of sensor is free of many of the restraints observed in other types of remote *Varroa* monitoring, proven through its success in capturing signals from both the entire honeybee colony and individual vibrations produced by single bee individuals^39–43^. Unlike gas sensors and microphones, accelerometers can reside in the hive long-term, and remain fully functional without the need for protection from propolisation. Additionally, due to the small size of the sensors (1000 mm^3^), and the fact that the colony readily builds normal comb over them (often brood filled), it appears that there is little effect, if any, of accelerometer presence on bees.

A major challenge of remote colony monitoring could be data bias resulting from short-term measurement periods^32^. Accelerometers overcome this as they record long-term (years), providing highly meaningful, continuous hive data^39–43^. In utilising accelerometers, a dataset containing a large number of naturally occurring vibrations can therefore be accrued, enabling the identification of patterns and trends, as well as the pinpointing of early warning signs for hive events such as swarming^40–43^. Accelerometers are therefore an ideal device for gathering non-invasive colony measurements, although they do presently remain expensive and unaffordable for any beekeeper.

We have previously demonstrated that accelerometers can detect the minute vibrations of individual *Varroa* mites, and from this data have carefully and meticulously characterised the features of specific mite pulses^44^. In the research presented here, we build upon our original characterisation of individual mite walking pulses, producing the first ever description of *Varroa* gait vibrational features. We then build a training database that exploits a highly effective feature of these vibrations, to be used as part of a classification system for identifying mite presence in capped brood, where mites spend 50% of their life cycle^1^. We report that the unique vibrational trace of *Varroa* gait can be used to discriminate between a mite on brood-comb and a bee emerging from her cell, as these honeybee vibrations were prominent in our recordings of capped comb. In doing so, we show that our discrimination software is capable of correctly classifying a mite vibration based on the uniqueness of its two-dimensional-Fourier transform. These exceptional features are further demonstrated through a second discrimination analysis of *Varroa* gait and that of two other invertebrate species (woodlouse and carabid beetle). We acknowledge some of the current limitations to our approach and offer future solutions. This research provides a significant step forward in *Varroa* biotremology as well as remote mite detection in colonies.

## Results

### Characterisation of Varroa gait features

**Figure 1:**
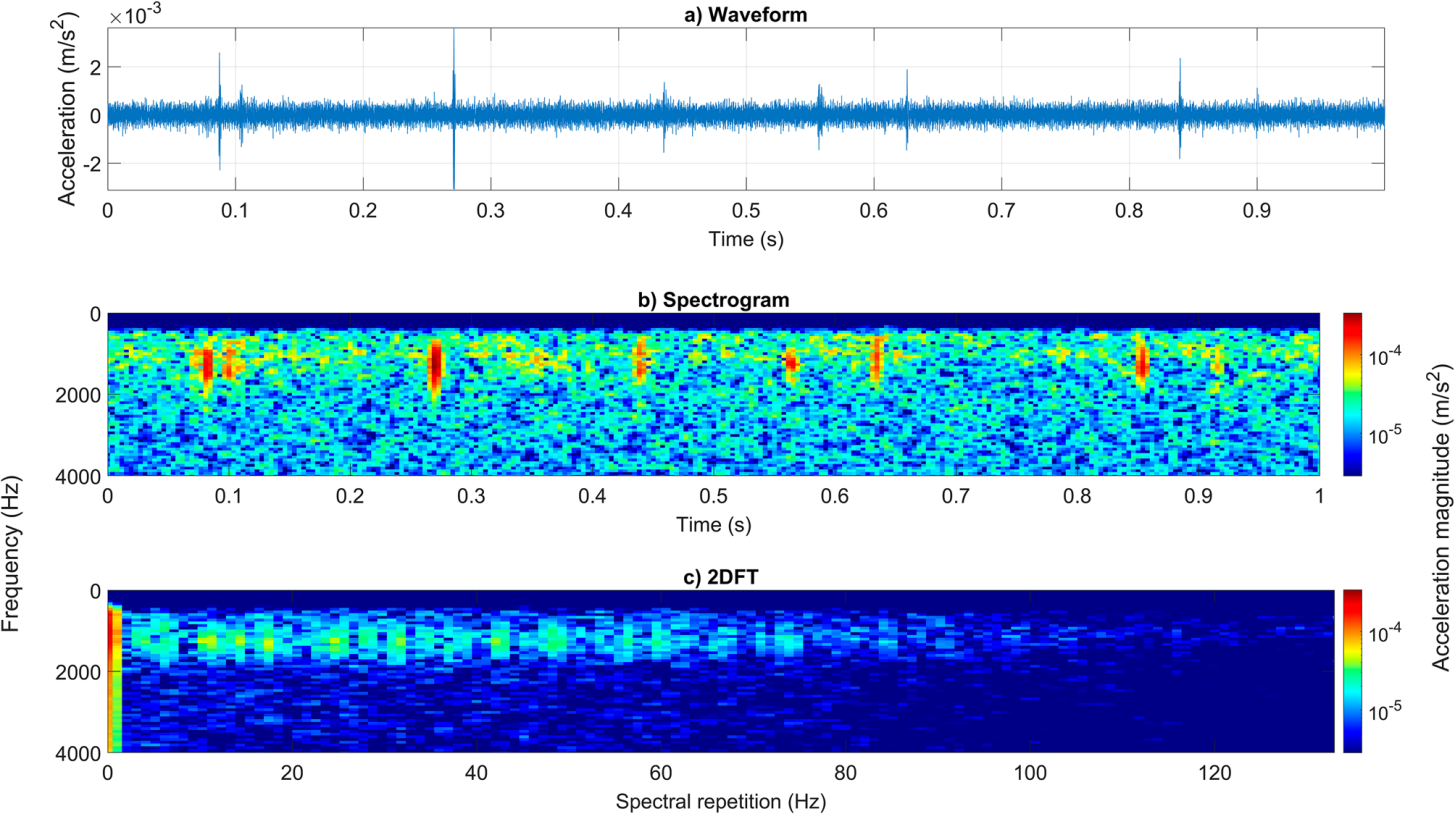
A representative example of 1 s of *Varroa* walking vibrations on brood-comb, viewed as an accelerometer waveform (panel ‘**a**’), spectrogram (panel ‘**b**’), and 2DFT (panel ‘**c**’). Magnitude of acceleration is logarithmic (to the base 10) in ‘(**b**)’ and in ‘(**c**)’, where dark red is the maximum (3.4 × 10^–4^ m/s^2^), and dark blue is the minimum (here forced to be 1/120 of the maximum). The spectrogram and 2DFT panels have been scaled identically and cropped to remove redundant frequencies (high pass filtered 0–500 Hz, cropped to remove frequencies above 4 kHz).

The unique vibrational pattern that constitutes the gait of a *Varroa* mite walking on brood-comb is here identified and characterised (see Fig. 1). Walking most often occurs in short bursts that last 1–2 s, and therefore a feature time duration of 1 s of the recording was deemed ideal for allowing suitable characterisation of the vibrations. Honeybee emergence vibrations occurred more continuously and are more variable than mite pulses in terms of frequency spectrum features (see supplementary Fig. 1).

*Varroa* mite walking, and honeybee emergence vibrations are easily identifiable via critical listening of the recordings (see supplementary Video 1). When viewed as 2DFTs, clear discriminatory features can be seen between the two types of vibration, alongside the third category of background noise (the reader is strongly advised to view supplementary Video 1 before reading any further), which was also separately looked into due to the poor signal-to-noise-ratio (SNR) that *Varroa* signals exhibit. The 2DFT image reveals both the fundamental frequency and any frequency harmonics observed in the vibration along the y axis, but in particular the visualisation of data as a 2DFT is remarkably suited to identifying repeating spectral signatures within a signal that exhibits a burst of periodic, similar pulses. Such a signal yields clear vertical traces on the 2DFT, providing straightforward reading of the spectral repetition of the vibration (along the x axis).

**Figure 2:**
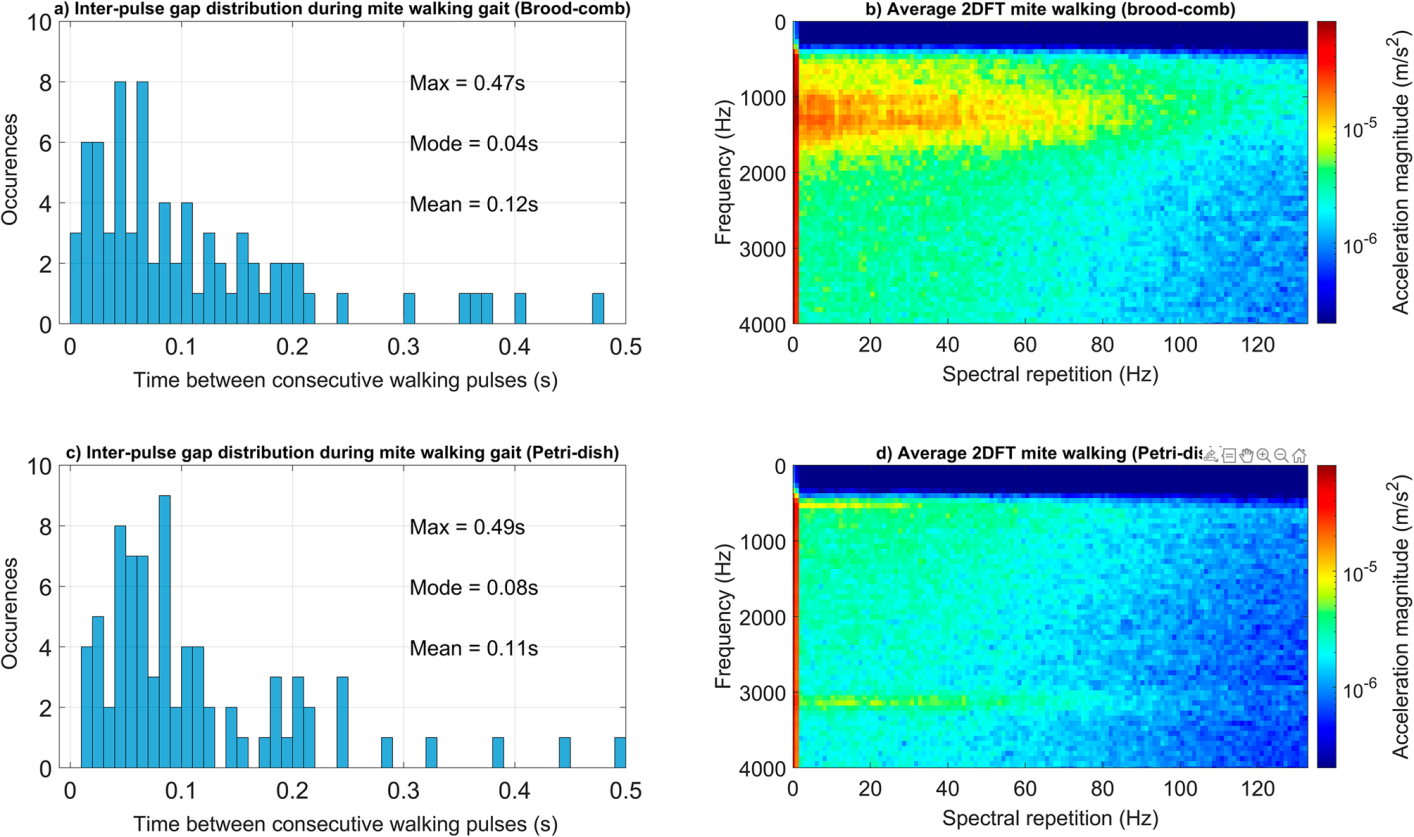
A histogram representing another type of quantification of mite walking pulses on brood-comb (panel ‘**a**’), and Petri-dish (panel ‘**c**’). The averaged 2DFT for walking pulses on each substrate is also shown (panels ‘**b**’ and ‘**d**’). In the histograms, time elapsed between two consecutive pulses is shown on the x axis. The data was obtained from 10 s of continuous walking behaviour on each of the two substrates. The Petri-dish serves as a ‘control’ to compare to the brood-comb, which is a less homogenous substrate. On the Petri-dish, the mite walks unhindered by any environmental obstacles such as substrate edges, bumps, and raised areas, and so provides clear quantification of gait. The results from both substrates, however, demonstrate that *Varroa* locomotion is highly similar on brood-comb to that of a flat surface. In the averaged 2DFTs, a similar pulse spectral shape can be seen up to 80 Hz over the horizontal axis for both Petri-dish and brood-comb. Magnitude of acceleration is logarithmic (to the base 10), where dark red is the maximum (9 × 10^–5^ m/s^2^) and dark blue the minimum (forced to be 1/400 of the maximum). Both 2DFTs have been scaled identically.

The honeybee vibrations residing in the recordings in our study did not typically demonstrate specific recurring frequency components (see supplementary Fig. 1). Mite walking vibrations, in contrast, exhibited repeated components that were more regular. Ten 2DFT images, each comprising of 1-s of *Varroa* walking vibrations from the recordings on both brood-comb and Petri-dish (a flat, homogenous surface, for comparison), were scrutinised. On both substrates, multiple spectral repetition frequencies were seen in each 2DFT, within a range between 4 and 60 Hz (see Fig. 1, see supplementary Fig. 2). Within this frequency range, 4 Hz (60%), 14 Hz (40%) and 6 Hz (30%) were seen most commonly on the brood-comb (some 2DFTs exhibited two strong peaks, hence the percentage total equalling 130%), whereas on the Petri-dish, more frequency variation occurred, with only 7 Hz (30%) observed more regularly. This feature is further highlighted when viewing the average 2DFT for each substrate, where frequency peaks are less identifiable on Petri-dish, due to the larger variation observed (see Fig. 2, panel ‘d’). The averaged 2DFT images show similar horizontal pulse spectral shape up to 80 Hz in the observed vibrational traces seen at 1000–1500 Hz (brood-comb) and 500 and 3000 Hz (Petri-dish) demonstrating that *Varroa* gait are comparable on both substrates (see Fig. 2, panels ‘b and ‘d’). It is known that mite vibrational traces vary in frequency features (vertical axis of the 2DFT) dependent on substrate vibrational modes^44^.

A second, independent method of gait quantification was then carried out, where the time elapsed between two consecutive walking vibrational pulses was identified for 10 s of *Varroa* walking behaviour on (i) brood-comb and (ii) Petri-dish. This demonstrated that 0.04 to 0.08 s is the mode time duration between successive pulses on both the flat Petri-dish and irregular brood-comb substrates (see Fig. 2). Note that there is a discrepancy between the common spectral repetition rates observed in the 2DFTs and the time intervals between consecutive walking vibrations (only 0.07 s matches the 14 Hz frequency component observed in the 2DFTs), due to the independent methodological differences. The 2DFT strongly emphasises periodicity in the pulses, whereas this information is deteriorated in the time analysis, as the intervals were quantitated between consecutive pulses, as is now further explained. Each walking leg produces its own revolutionary cycle as the *Varroa* moves, and as it was unknown which leg caused which vibrational trace, the pattern of this cycle can be lost (the time interval between two pulses produced by the same leg cannot be known). The 2DFT is capable of extracting and revealing this periodicity, whereas consecutive pulse interval analysis is useful for establishing how quickly the legs move.

By comparing Petri-dish and brood-comb walking data, we have been able to confirm gait vibration feature similarities regardless of substrate. Our brood-comb data was limited by the number of mites seen walking and the fact that the behaviour was in short bursts (1–2 s) on this substrate in comparison to Petri-dish, where (in our experience) mites walk uninterrupted for prolonged periods.

A few seconds of *Varroa* gait data is insufficient to allow the identification of features that are truly generic to this mite. Therefore, to further substantiate the identification of unique features of the vibrations resulting from the gait of a *Varroa* and support the walking data that we have used for our detection analysis, we also compared the vibrations of mites walking with two other invertebrates also walking on Petri-dish.

**Figure 3:**
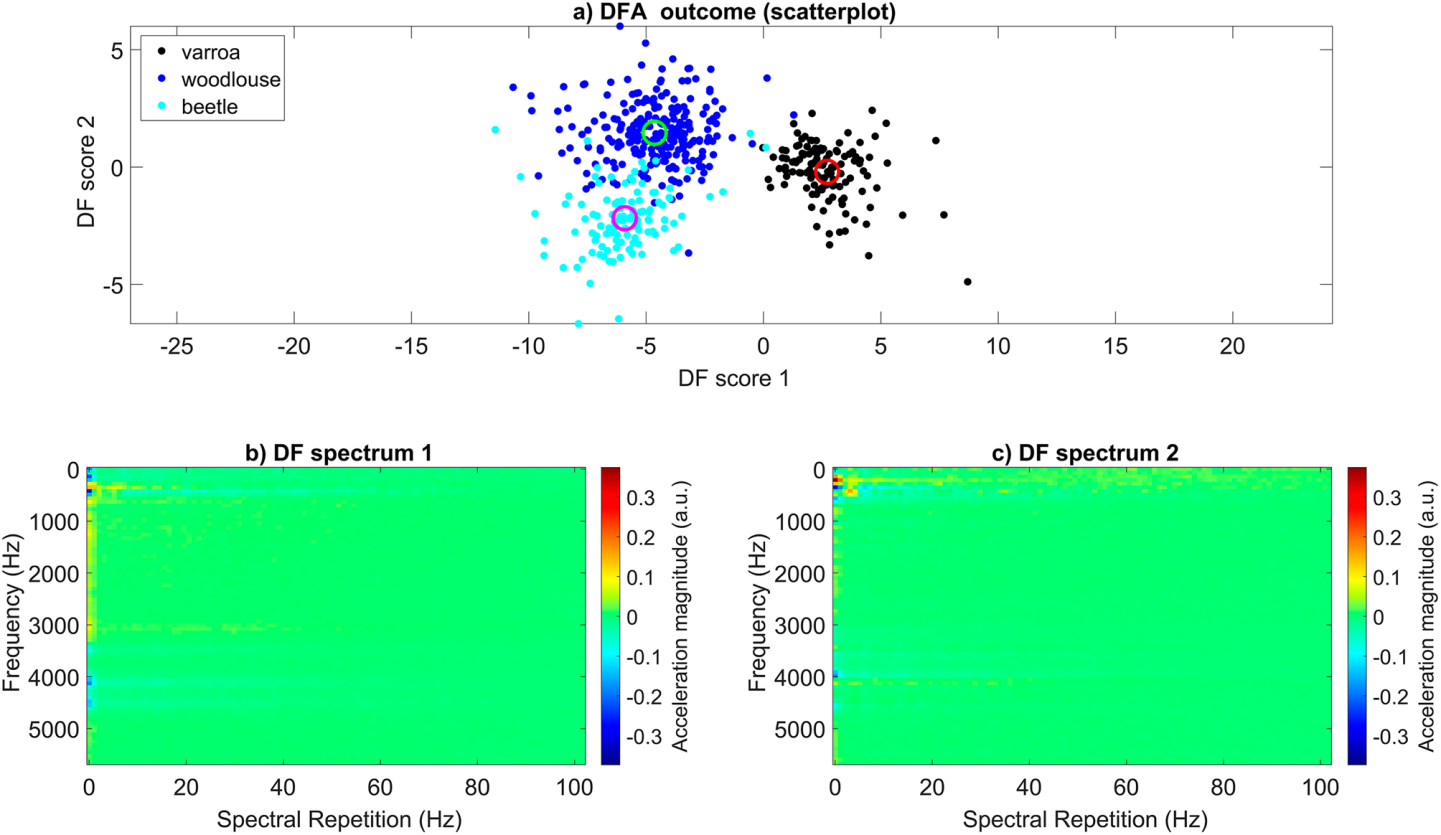
The results gathered from machine learning on the invertebrate gait TDB. (Panel ‘**a**’) exhibits the scattering of mite (black), woodlouse (blue), and beetle (cyan) data in DF space. The centroid for each data group (mite = red, woodlouse = green, beetle = magenta) is also shown using a hollow circle. All categories demonstrate very good discrimination. (Panels ‘**b**’ and ‘**c**’) show images (discriminant 2DFTs) generated from the PCA/DFA that were then used as reference images in cross-correlation product analysis. Magnitude of acceleration is in arbitrary units, where dark red and dark blue indicate features of the 2DFT that have the strongest influence on the discrimination between the three groups. Green is indicative of zero, i.e., those features that do not contribute to the discrimination.

A training database (TDB) was created that contained 1-s long extracts of three invertebrate species (6 *Varroa* individuals = 125 s, 3 woodlouse individuals = 239 s, 3 carabid beetle individuals = 113 s) walking on Petri-dish (the reader is strongly advised to view supplementary Video 2 before reading any further). A simple machine learning (principal component analysis (PCA)/discriminant function analysis (DFA)) algorithm was used to search this database for components that exhibited high variation, and the three species demonstrated clear clustering in DF space when using only 17% of all deviations (see Fig. 3, panel ‘a’). Some marginal, unavoidable (as the overlap occurred regardless of the percentage of deviations included) overlap can be seen between the woodlouse and beetle data (see Fig. 3, panel ‘a’). Two discriminant 2DFT images were also produced from this analysis, to visualise the feature variation that allowed the high-quality clustering in the dataset (see Fig. 3, panels ‘b’ and ‘c’). The colour-coding of the discriminant images is the quantification of the 2DFT features in each category that vary from one another. Discriminant 2DFT no.1 and no.2 respectively highlight the variation linked to the horizontal and vertical axes of DF score space, where the areas of highest variation can be seen mostly in red and blue (see Fig. 3, panels ‘b’ and ‘c’). Both discriminant images demonstrate high frequency harmonics (3500, 4000, 4500 Hz) that were observed in the 2DFTs of woodlice and beetles, but not *Varroa* (highest frequency band 3100 Hz), indicating that these are strong discriminatory features along both dimensions. Additionally, discriminant 2DFT no.2 has a strong spectral repetition feature at approximately 4 Hz which contributed to the variation between categories.

The scatterplot that illustrated the outcome of group clustering and the discriminant 2DFTs (see Fig. 3) were then used for further analysis, to test the ability of the machine learning to discriminate mite from woodlouse and beetle in recordings that did not contribute to the training of the computer. To do so, extracts of the three types of invertebrate walking were plotted onto DF space following cross-correlation product analysis between the 1-s excerpts of the recording and the two discriminant 2DFTs. The outcome was highly successful, with a high percentage of points falling into the correct area of DF space (7/10 tested mite recordings =  > 80% points falling into the mite area of DF space), as dictated by the scatterplot (see Fig. 3, see supplementary Table 1). When observing the synchronised movements and vibrations of each animal, the success of the machine learning algorithm can also be seen (see supplementary Video 2), where the walking vibrations always fall in the vicinity of the correct scatterplot centroid.

To further substantiate the unique features of *Varroa* gait, analyses was carried out to demonstrate the speed of mite walking. Average *Varroa* velocity was calculated using 10 s of video of a mite walking on Petri-dish. Maximum velocity was found to be 4 mm/s, with an averaged mode speed of 2 mm/s when walking at a steady pace (see supplementary Fig. 3).

**Figure 4:**
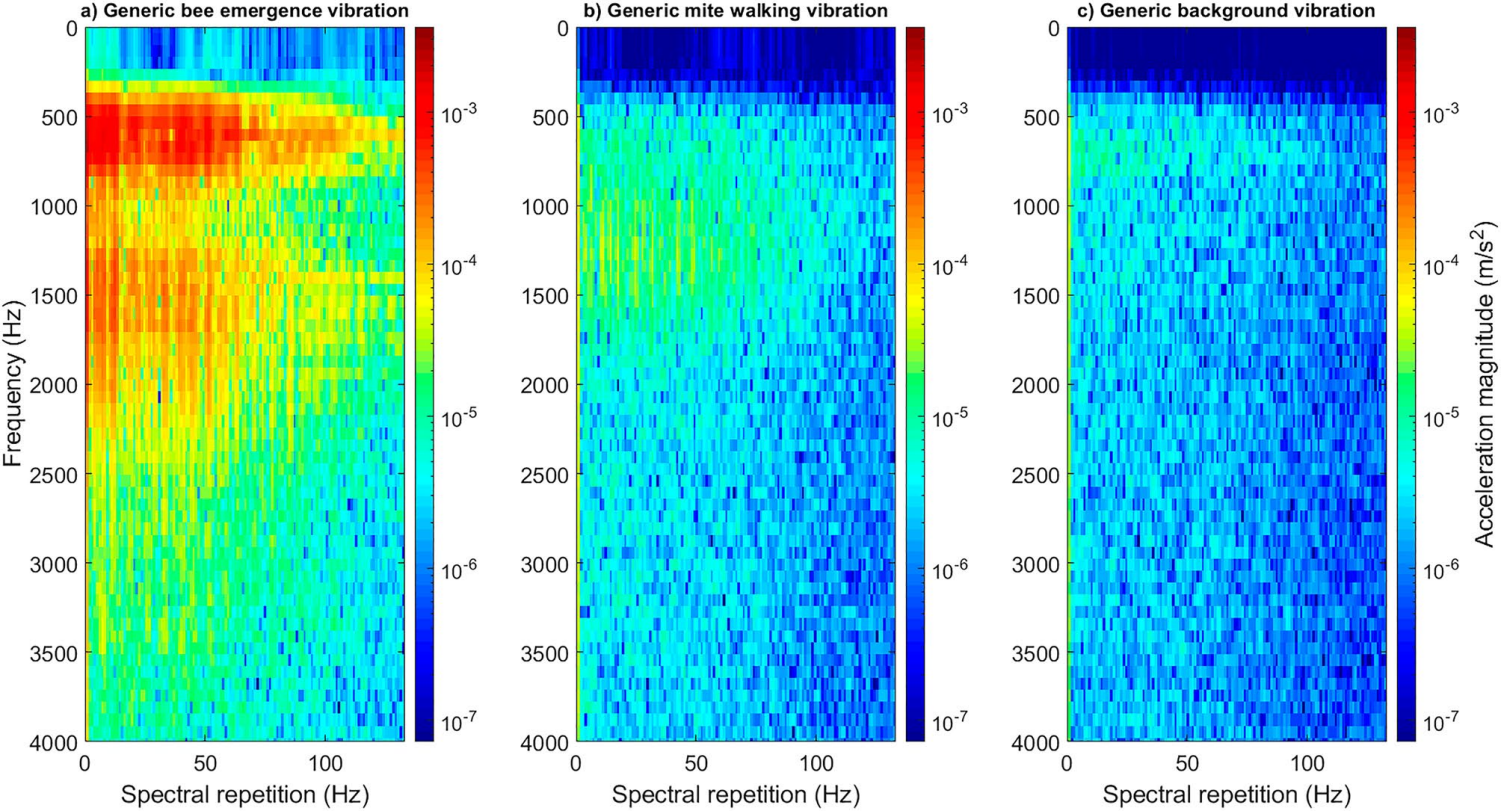
Representative 1-s-long extracts of honeybee (panel ‘**a**’), mite (panel ‘**b**’), and background (panel ‘**c**’) vibrations, viewed as 2DFTs. The differences between the three types of signals can be seen here. The commonly occurring mite spectral repetition frequency can be seen around 4–6 Hz on the x axis (panel ‘**b**’) and can also be more clearly seen in Fig. 1, panel ‘c’. All accelerometer data has been high pass filtered (0–500 Hz) to reduce the impact of background vibrations on mite traces in particular. No traces of interest were identified beyond 3500 Hz. Magnitude of acceleration is logarithmic (to the base 10), where dark red is the maximum (3.6 × 10^–3^ m/s^2^) and dark blue the minimum (forced to be 1/50,000 of the maximum). All three panels have been scaled identically.

Returning to the honeybee and mite analysis, differences between the two species’ vibrations were not only observed in spectral repetition rate, but also in pulse spectral shape. Mite walking vibrations mostly occurred within one frequency band, between 500 and 2000 Hz (see Figs. 1 and 4), whereas honeybee vibrations generally exhibited two separate bandwidths, between 500 and 900 Hz, and 1250 and 2100 Hz (see Fig. 4, see supplementary Fig. 1). Background vibrations, as expected, lacked any clear traces (see Fig. 4) as these are comprised only of the ambient noise experienced within the sound-isolated room in which the brood-comb recordings took place. This indicates that the way the animals leg collides with the substrate it is walking on is also different between different species.

The differences between each category of vibration (mite, bee, background) can also be seen when viewing video recordings synchronously with their vibrational data (see supplementary Video 1). Mite and background 2DFTs are easily distinguishable from those of the honeybee category, but due to poor SNR, which is typical of *Varroa* mite vibrations, there are fewer strong discriminating features between the 2DFTs of mite and background (see supplementary Fig. 4).

### Creation of a training database to identify variation between mite walking, honeybee emergence and background vibrations within brood-comb samples

**Figure 5:**
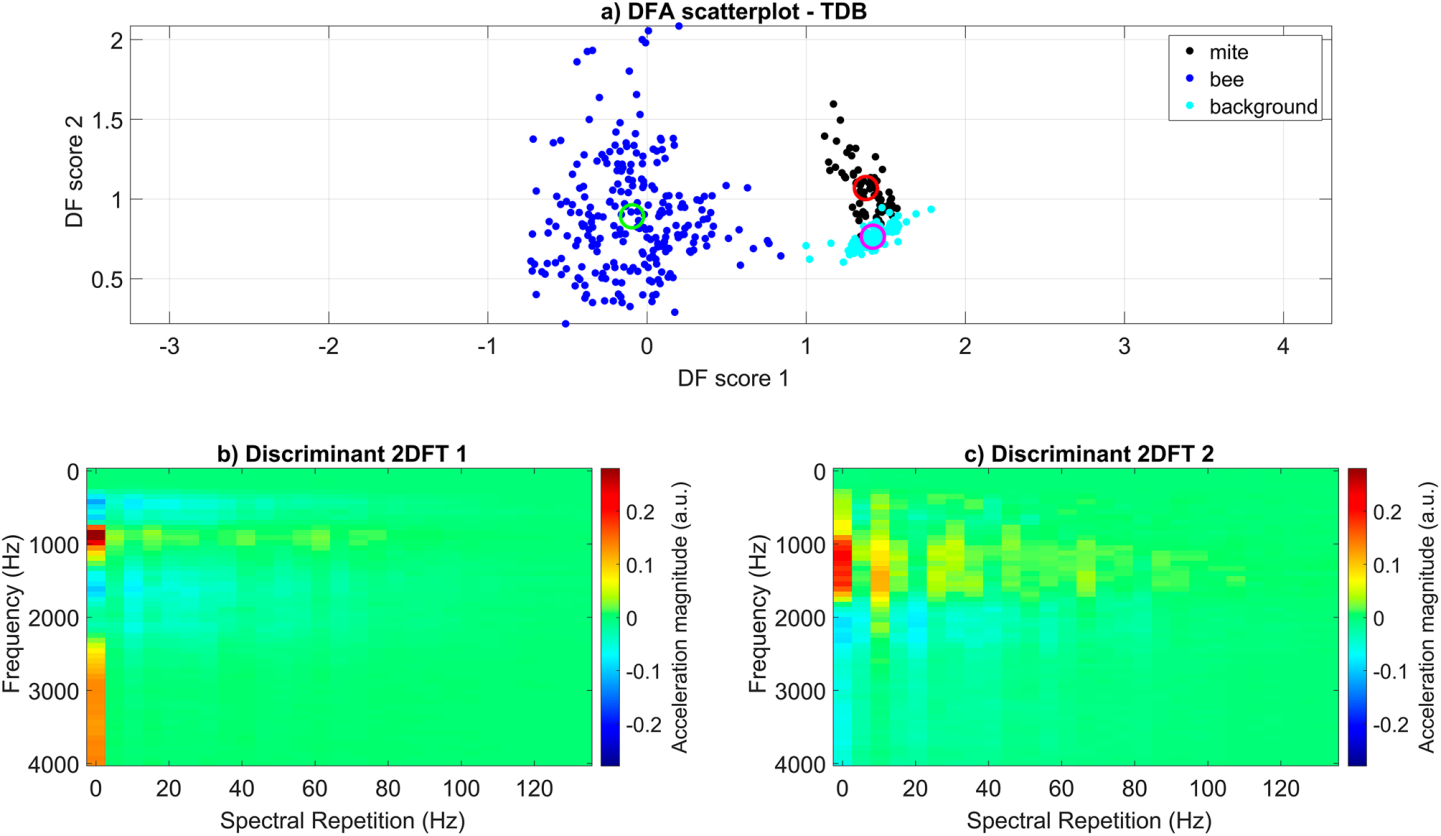
The results gathered from machine learning on the mite, honeybee, and background TDB. Panel ‘(**a**)’ exhibits the scattering of bee (blue), mite (black), and background (cyan) data in DF space. The centroid for each data group (bee = green, mite = red, background = magenta) is also shown using a hollow circle. All categories demonstrate very good discrimination. Panels ‘(**b**)’ and ‘(**c**)’ show images (discriminant 2DFTs) generated from the PCA/DFA that were then used as reference images in cross-correlation product analysis. Magnitude of acceleration is in arbitrary units, where dark red and dark blue indicate features of the 2DFT that have the strongest influence on the discrimination between the three groups. Green is indicative of zero, i.e., those features that do not contribute to the discrimination.

A separate TDB was created that contained 1 s excerpts of mite, bee, and background vibrations (see supplementary Fig. 4). The same analyses techniques as used for the invertebrate walking discrimination were then implemented for this entirely separate TDB. The three groups demonstrated clear clustering in DF space when using 25% deviations (see Fig. 5, see supplementary Video 1). Some negligible overlap between the mite and background data can however be seen (see Fig. 5), as expected from the already mentioned low SNR of the *Varroa* vibrational data.

Sections of discriminant 2DFT no.1 that are in colours representative of high variation (blues/reds) closely reflect that of a honeybee 2DFT, where the spectral repetition axis lacks specific frequency peaks. Independently, 2DFT no.2 shows stronger variation (in orange) at a spectral repetition frequency of 7–13 Hz, close to that commonly seen in mite 2DFTs, but not honeybee or background, and therefore is the strongest feature that discriminates the categories along the vertical dimension (see Fig. 5).

The scatterplot that illustrated the outcome of group clustering and the discriminant 2DFTs (Fig. 5) were then used for further analysis, to test the ability of the machine learning to discriminate mite from honeybee in recordings that did not contribute to the training of the computer.

### Classification of data within recordings that contributed to the training database (TDB)

**Figure 6:**
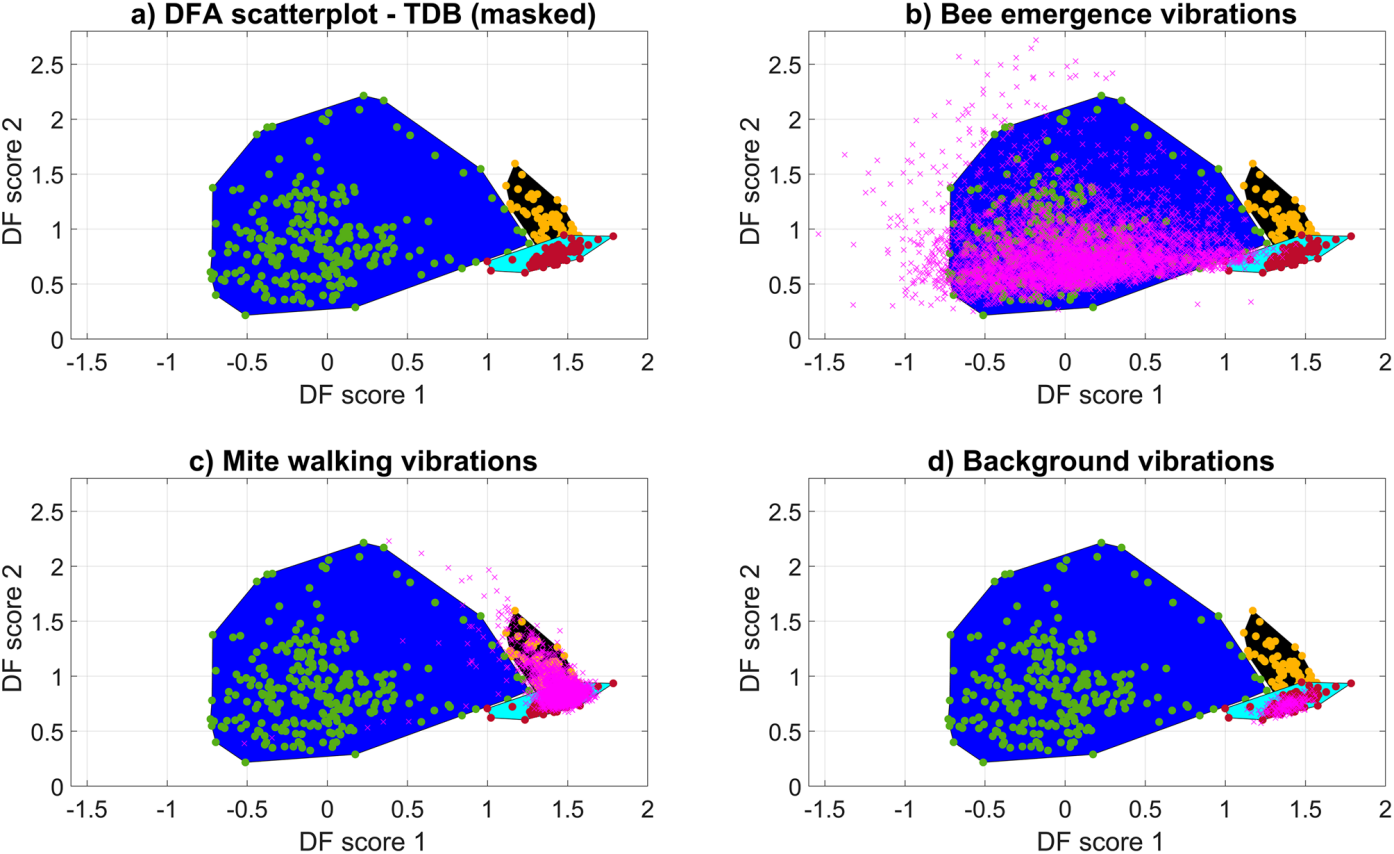
The TDB scatterplot (panel ‘**a**’) and the outcome of projecting data onto the same DF space (panels ‘**b**’, ‘**c**’, and ‘**d**’). Panel ‘**a**’ shows the TDB scatterplot with the area of each cluster further highlighted as a filled polygon (blue area = bee, black area = mite, cyan area = background). The peripheral datapoints for each group (bee, mite, background) were used to define the coloured areas, and the original points for each cluster can be seen as dots within each mask (bee = green, mite = yellow, background = red). The other panels (‘**b**’, ‘**c**’, and ‘**d**’) each show additional data (pink crosses) from carefully selected time periods within the recordings that contributed to the TDB. Panel ‘(**b**)’ shows 20 min of ‘bee only’ vibrations, panel ‘(**c**)’ 12 min of ‘mite only’ activity and panel ‘(**d**)’ 50 s of ‘background only’ noise. Note that as the mite walked intermittently in short bursts of 1 s, it was not possible to omit background vibrations completely from this category.

The full recordings from which excerpts were used to create the TDB (see supplementary Fig. 4) were first tested in this analysis. As the recordings were long (30–120 min), not all the vibrations found within them were used in the TDB formation (bee data used = 242 s, mite data used = 66 s, background data used = 87 s). It was necessary to start by establishing whether the remaining, known vibrations in these datasets could be successfully classified using the first training algorithm. The entire accelerometer track for each recording was therefore projected onto DF space (see Fig. 6) for categorisation purposes.

The performance of the classification analysis for all these recordings was very high, with the datapoints falling into the correct masks based on the known vibrations occurring in each (see Fig. 6). It is worth noting that ‘bee only’ and ‘mite only’ sections did still contain some background noise, as areas of recordings containing these activities are inherently, occasionally also comprising of pure background signal.

When considering the full duration of each recording, a negligible percentage of points fell into the incorrect mask e.g., a ‘honeybee only’ recording exhibited a small percentage of points in the *Varroa* mask (honeybee recording 1 = 0.07% of datapoints overlapping into the mite mask, honeybee recording 2 = 0.01% of datapoints overlapping into the mite mask, mite recording 1 = 0.7% of datapoints overlapping into the honeybee mask, mite recording 2 = 5% of datapoints overlapping into the honeybee mask) (see supplementary Table 2). In *Varroa* recordings, background noise was prominent as mites were seen to walk less frequently, and in shorter bursts than observed in honeybees producing vibrations (see supplementary Table 2).

The outcome of the automated discrimination for each recording was further scrutinised via critical listening and observation of the corresponding video data to determine the classification success. In light of the very simple machine learning algorithm used here, and the relatively small size of the training database, the performance achieved is truly excellent, and further demonstrates the high specificity of the 2DFT to the information of interest.

### Classification of data within recordings that did not contribute to the TDB

Following this success, separate recordings that were not used in the formation of the database were then tested with the same algorithm. The samples used for these recordings each consisted of a section of capped brood-comb that was removed from a honeybee colony, and immediately placed in a sound-isolated room to be recorded both by an accelerometer and a camera, synchronously. Following this, these samples were then dissected to quantitate and establish whether *Varroa* mites were present within them or not. Eight recordings of specimen that contained *Varroa*, and eight that did not contain *Varroa* were then analysed in the same way as all previous recordings, using the same machine learning algorithm. However, it must be noted that it was not possible to gain any corroborative evidence that the mites residing in the opaque capped cells of these samples were active during the recording period or not.

Unfortunately, the discrimination outcome for all 16 recordings was poor. Data projection varied from sample to sample, with no clearly identifiable features or differences between the *Varroa* present and *Varroa* absent categories (see supplementary Fig. 5). For most recordings, the data landed mostly over the honeybee and background masks, with negligible overlap into the mite mask (see supplementary Fig. 5, see also supplementary Table 3). One recording further demonstrated anomalous results, where the major area of clustering occurred over the mite mask, despite the fact that the recording originated from a specimen known to lack *Varroa* (see supplementary Fig. 6).

This recording was scrutinised in more detail, to explore whether improvements could be made to the training to enhance its success even with the anomalous recording.

### Investigation into the anomalous recording

Upon critical listening of this particular *Varroa* absent recording, many honeybee vibrations were heard that exhibited features similar to those of *Varroa* walking (see supplementary Fig. 7). Both types of vibratory trace occurred between 500 and 2000 Hz, with similar patterns of production over a 1 s time window (see supplementary Fig. 7). However, when critically listening to the vibrations produced by this bee, and a mite walking without visually inspecting the 2DFT images, 9/10 samples could be correctly identified.

Following the success of the critical listening task, it was deemed worthwhile to attempt feature discrimination between four, instead of three, categories: this anomalous recording and the original three categories in the TDB.

The TDB with four categories produced clouds of points exhibits strong between-category overlap in DF space. However, 50% of the *Varroa* data did cluster separately, showing that it was possible to discriminate between the anomalous honeybee and some mite vibrations, regardless of the initial similarity observed between their vibratory traces (see supplementary Fig. 8), and in good agreement with the promising discrimination achieved subjectively by critical listening.

When testing the long-term brood-comb recordings that either did, or did not, contain *Varroa* using this updated algorithm, the results still indicated a lack of features unique to each group (*Varroa* present or *Varroa* absent). The number of datapoints from these recordings that were observed to land in the ‘mite-only’ area of the mite mask, i.e., the area of the mask that did not overlap with the anomalous bee mask, was unfortunately negligible for both '*Varroa* present’ and ‘*Varroa* absent’ recordings (see supplementary Table 4, see also supplementary Fig. 8).

Average background removal from each of the recordings, and an increase in time duration of the accelerometer extracts that contributed to the TDB (from 1 s to 1.5 s and 2 s), also did not improve the discrimination outcome any further, unfortunately.

## Discussion

The unique vibrations resulting from the gait of a *Varroa* have here been characterised, demonstrating the two-dimensional-Fourier-transformation feature as being remarkably well suited to its automatic and highly specific recognition. Previously, mite walking pulses have been discussed mostly in terms of frequency and magnitude of acceleration^44^, and the work carried out as part of this study has now provided additional, distinctive information on their features.

Although one specific bee recording of ours exhibited remarkable similarities to that of *Varroa* walking pulses, it was possible to retrain our algorithm and retain our claim that we have identified a vibration feature highly specific to *Varroa* gait. When critically listening to audio samples of this bee against that of a walking mite, 9 out of 10 could be correctly recognised, indicating that there are features that define one type of vibration from the other. Additionally, when adding the vibrations of this bee to the TDB, discrimination could still be found between this category and 50% of mite walking vibrations, further confirming their differences. The unexpected *Varroa*-resembling bee vibrations were found in just 1 of 16 recordings, with no other exhibiting unusually similar vibrations to a mite walking.

The highly successful outcome of the invertebrate discrimination further demonstrates that *Varroa* gait is highly different to theirs, and combined with its unusual anatomy^2^, provides tantalising indications that it may even be unique. Interestingly, both the woodlouse and beetle recordings could be discriminated from one another, but not as well as they could from mites, demonstrating that, for our study, gait vibrations are highly species specific for *Varroa* but not as much for woodlouse and beetle, despite the vast difference in appendage number (beetle = 6 legs, woodlice = 14 legs).

### A comparison of 2DFT and pulse interval analysis in the identification and discrimination of Varroa gait features

Our results showcase the previously unknown features of *Varroa* gait vibrations through 2DFT and pulse interval analysis. The strength of the 2DFT in the identification and discrimination of *Varroa* gait has particularly been highlighted. Pulse interval analysis required at least 10 s of mite walking data to identify time elapsed between consecutive pulses, and therefore speed of movement. The 2DFTs, on the other hand, were created using just 1 s of walking recording, and revealed more accurate information. This is because pulse interval analysis relies heavily on each *Varroa* step producing a detectable vibrational trace. It is known that a percentage of vibrational pulses will not be picked up by the accelerometer, as mites will not produce vibrations at a consistent strength^44^. The 2DFT makes up for this discrepancy, ‘filling in the blanks’ where vibrational data was not captured, therefore providing a more accurate representation of gait features.

Although not as successful as the 2DFT, pulse interval analysis was still a useful tool for confirming that our recordings of mites walking on brood-comb showed genuine *Varroa* gait patterns because (i) our brood-comb recordings comprised mostly of short bursts of activity (1–2 s), and (ii) the brood-comb is an irregular surface. Pulse interval analysis using Petri-dish data (a homogenous and flat surface, i.e., potentially less cumbersome for mites to locomote over and therefore suited for capturing gait features), and longer recordings (10 s each on brood-comb and Petri-dish), demonstrated that walking pulses are produced by mites rapidly, with an elapsed time of 0.04–0.08 s between consecutive vibrations on both types of substrate. This indicates that *Varroa* gait is not disadvantaged by the irregularity of the brood-comb surface. The analysis, alongside that of the invertebrate discrimination which tested the gait of 10 mite individuals, has helped to confirm that the brood-comb recordings used in the training data were representative of natural locomotion in *Varroa*, even though the mites did not move for long durations.

The 2DFT and pulse interval results, alongside the velocity analysis, also confirm former anecdotal descriptions of *Varroa* locomotion that report the behaviour to be fast and efficient^2,8^. We have affirmed that *Varroa* are capable walkers, regardless of media, with the first quantification of a common observation that *Varroa* can move rapidly, which appears to be their natural locomotory style. This is an adaptation that must benefit the life cycle of the mite, as they need to move around on various substrates and remain safe from honeybees (e.g., the adult bee body where mites move between the abdominal tergites to feed and also avoid removal by bees^45,46^, and the wax cell which they must navigate around to defecate, reproduce and avoid being squashed by the spinning larvae^47,48^.

### The benefits of 2DFT analysis as highlighted by comparisons to spectrogram analysis

Previously viewing individual mite walking traces as spectrograms provided the first perspective of their typical vibrations, disclosing the absolute timings of the vibrational pulses originating from the individual steps^44^. In looking at a group of walking pulses as 2DFTs, we have here contributed an alternative viewpoint where the pattern of the time course of the successive pulses is shown, rather than their absolute timings. The 2DFT is not only offering novel representation of a *Varroa* mite vibration but it is also found to be a powerful feature of the gait, permitting its high-performance numerical discrimination. The use of spectrogram images was sub-par in comparison to that of 2DFTs, because in these, the absolute timings of the gait pulse is retained as an important, although irrelevant, signature in such analysis. In this case, the 2DFT provides a better gait feature, i.e., one that is not influenced by anything other than changes made by the animal, such as locomotory speed. The inclusion of absolute time in spectrogram images means that the pulses seen in the image window will differ dependent on when they were produced in time (and therefore the pulse interval gait feature will differ between spectrograms). As discussed earlier, the 2DFT is free of this constraint and will demonstrate the same periodicity features regardless of absolute timings, so long as the mite retains consistent walking speed.

This led us to change our focus from the spectrogram to the 2DFT in our detection investigation. Previously, in the field of bioacoustics, 2DFT images have only been the focus for (i) the honeybee dorso-ventral abdominal vibrations and (ii) honeybee colony swarming vibrations^42,43^. The results achieved in this study therefore expand the repertoire of 2DFT use in vibration feature analysis and invertebrate vibro-acoustic detection, which until now systematically relied on waveform or spectrogram images^49–54^.

As mentioned, a quantitative feature of the 2DFT image is that the observed repeating frequency components will reflect the mite walking speed. This is most probably the reason why the recurring frequencies seen in the Petri-dish were not exactly the same as those in the brood-comb 2DFTs, as mites may have walked at a different speed dependent on substrate (frequency components were observed within a range between 4 and 60 Hz on both substrates, but there were spectral repetition features identified on the brood-comb that were commonly seen, whereas on Petri-dish these varied more substantially). It may also be that because the Petri-dish is a substrate foreign to the *Varroa*, it may alter its gait, accordingly, as has been seen in other invertebrates (cockroaches^55^, caterpillars^56^, tardigrades^57^), which may have led to the variable frequencies observed. However, both brood-comb and Petri-dish 2DFTs showed excellent clustering in our discrimination analyses, suggesting that walking speed is consistent for each substrate. When considering velocity in the brood cells, the environment available is highly space limited^47,48^ and therefore mite locomotory speed should not vary too dramatically. As a result, 2DFT analysis should offer reliability in the detection of mite walking within the cells.

### Can Varroa walking vibrations be detected when mites are within the brood-cells?

When classifying honeybee, mite, and background vibrations from the recordings that partially contributed to the formation of the first TDB, the outcome was highly successful. Unfortunately, this was not the case when testing the completely independent set of brood-comb recordings, most probably because: (i) the vibrations originating from *Varroa* mites within capped brood cells are severely attenuated, and (ii) *Varroa* displacements and activities are perhaps more scarcely produced than when they are exposed, i.e., outside of the brood cell with more space to move around. The results gained from this endeavour can now be used to identify problems and solutions for future experimentation.

At present, we are limited by the sensitivity of the accelerometer sensor used (1000 mV/g), as this is the most sensitive device available on the market for that size (1000 mm^3^) and mass (10 g). As a result of the miniscule size and mass of *Varroa* mites (1–2 mm, 0.42 mg), signal-to-noise-ratio (SNR) was relatively poor and probably had a detrimental effect on the performance of the discrimination algorithm. *Varroa* vibrations typically suffered from low SNR when compared to honeybee emergence vibrations, which can be seen when viewing all the contributing TDB data (see supplementary Fig. 4). This is to be expected when considering the mass of a honeybee (115 mg^58^) and comparing it to that of a *Varroa* mite (0.42 mg^44^). When compared to another *Varroa* mite vibration (the jolting), walking pulses also generally exhibited weaker magnitudes of acceleration^44^.

Additionally, the strength of the detected walking pulse may also be further reduced when originating from within the brood cells, in comparison to *on* the brood-comb, a drier and stiffer substrate. It was necessary to collect examples of mite walking in the latter scenario so that visual evidence could be corroborated with the corresponding vibrations. Although mites are known to move around regularly when within the capped cells, the free space available to them for movement is highly restricted^47,48^. It is likely that mite walking vibrations suffer under these conditions, particularly as they are perhaps dampened by the presence of soft tissue of the developing bee. We have previously investigated *Varroa* walking behaviour on soft-bodied, developing honeybee larvae, and found that any vibrations related to this are never detected using accelerometer sensors^59^. The mites in our study may also not have walked on the cell wall at all, instead remaining on the larvae/pupae, which would have likely degraded vibration propagation. Time spent on the cell wall varies dependent on the length of time that a *Varroa* has been in the capped cell, where individuals heavily reside on the larva following emergence from the larval food (0–6 h post capping), and periods spent on the cell wall then gradually increase over time^47,48^. As we had no visual evidence of the movements carried out by *Varroa* in the capped brood samples, the activity levels and the substrate that individuals resided on were unknown, which may have contributed to the lack of detectable mite-related vibrations in our study.

Walking pulses were focussed upon in this study as they are known to occur in the reproductive phase^47,48^. However, it would be useful to further investigate other mite behaviours that occur in the brood cell and identify if any produce measurable vibrations. Perhaps there is an alternate activity that can be better detected for the purpose of remote mite monitoring. For instance, we have previously identified *Varroa* jolting as a suitable candidate for remote mite detection^44^, although an initial investigation into whether mites produce this vibration when in the reproductive phase is first required. A second, detectable vibration produced by *Varroa* on the developing larvae, currently theorised to be ‘repetitive sampling’ behaviour^48^ has also been identified and somewhat characterised in terms of its vibrational features^59^, although this behaviour and its corresponding vibration also require some further analyses.

Alternatively, a different experimental approach could be taken in terms of exploring other locations for mite detection. In this study, we focused on the brood cells as the most logical starting point for our mite monitoring investigation, as 65% of individuals are expected to reside here at any time during the brood rearing season^60^. However, following the challenges faced in this initial exploration, and in the context of the very clear *Varroa* jolting pulses we have detected in a Petri-dish^44^, we would like to suggest an alternative location that may be more effective (we would not suggest the use of *Varroa* vibrations detected when they walk *on* the honeycomb, as this would occur very rarely, and if contact with the cell surface did occur, this would be only for a brief moment between leaving an adult bee and entering a larval cell^61^).

Accelerometer placement on the bottom boards of colonies show promise as part of the remote mite detection strategy. Beekeepers use boards specifically for the purpose of counting mites that fall from the hive, and in doing so can gain accurate estimations of *Varroa* populations^29,62^. Mites, in our own experience of counting individuals on boards, are often found alive, jolting, and walking around, demonstrating an exciting benefit to accelerometer placement in this area.

The measured vibrations would likely exhibit higher SNR, particularly as the board is largely free from the effect of the colony’s vibrations, as bees are not in direct contact with the substrate. This is unlike the brood-comb, which suffers not only from the vibrations of emerging bees, but also from those working on the comb externally. Furthermore, this substrate could be useful for jolting pulse capture, as we already know that mites produce this behaviour when placed outside of their usual environment (adult honeybees, or within capped brood)^1,44^. Jolting pulses are stronger than walking ones^44^, and this would not only benefit the specific detection of mites, but perhaps also further our understanding of the behaviours’ function.

## Conclusion

We have here discovered exciting, additional features of *Varroa* walking vibrations, revealed through the use of 2DFT image analysis, which demonstrates the advantages of this method in *Varroa* vibration research.

The 2DFT has great potential for future machine learning and automated detection based on the vibrational features of *Varroa* gait. Our work here has laid the groundwork for future investigation into its use as a remote mite detection tool. We have established that excellent discrimination can be achieved when testing known accelerometer recordings, and although this method was less successful when examining independent recordings using *Varroa* in capped brood-cells, we believe that this was due to the enhanced signal attenuation, and the small size and potentially low activity levels of the *Varroa* mites within our capped brood samples. In order to make *Varroa* signals detectable with accelerometers, we suggest novel potential solutions to promote remote mite monitoring using accelerometer sensors. Importantly, this work has yielded the further characterisation and understanding of *Varroa* walking vibrations, which can benefit future management strategies and add to our knowledge of mite biology.

Remote honeybee colony monitoring is on the rise^32^, and research into innovative methods such as vibration capture can only serve to support its improvement. For example, previous work using accelerometers has enabled the capture of important physiological honeybee activities such as the dorso-ventral-abdominal signal^42^. Particular focus on the detection of important threats such as *Varroa* infestation is critical for the maintenance of healthy hives, our research offers a novel approach to this problem, which we will now continue to work on and improve, based on the investigations that we have carried out so far.

## Methods

### Data acquisition

#### Collection of mite vibrations

To enable successful *Varroa* mite waveform classification, a database of known vibrational specimen was built for training purposes. Mite vibrations were acquired from data collected in our previous study^44^, where active mites were collected from the bottom-board of a honeybee hive, then placed on samples of brood-comb (approximately 3 × 3 × 2.5 cm^3^) for their behaviours to be observed and recorded. The vibrations that corresponded to mite walking and jolting behaviour were identified, extracted and characterised in terms of their specific vibrational features^44^.

For this present study, a collection of mite walking pulses was chosen from this same data, based on their clarity within the accelerometer track, to ensure that the training database would be built using highly reliable examples of the vibration in question.

#### Collection of other vibrations present in brood-comb

It was necessary to include in the training database other occurrences of vibration, to demonstrate whether pulses of mites could be successfully discriminated against alternative brood sample noise.

To do this, 18 sections of capped brood-comb of the same size were removed from colonies of *Apis mellifera* using a scalpel. These were transferred to the laboratory, where each sample was individually recorded using the same video camera and accelerometer attached to a Petri-dish as outlined previously in Hall et al., (2021)^44^. Video and vibrational data were collected for each sample over long-term periods of 30–120 min. Following this, the sample was dissected to determine the number and age of each developing bee within. Any *Varroa* mites present in the sample were also counted (samples containing mites = 8, samples lacking mites = 10).

In many of the samples, ‘scratching’ vibrations were identified by critical listening. In one sample, these vibrations were visibly attributed to a bee chewing at the capping of her cell and emerging. This was the only instance where a bee was seen exiting a cell, but ‘scratching’ vibrations were common in all capped brood-samples. As a result, we could not directly link all these vibrations to bee emergence activities, but could identify that they were made by bees, as these were the only species (alongside *Varroa*, which were found in half of the specimens) found in the samples. Short periods of “no vibration”, i.e., background noise, also occurred.

#### Collection of invertebrate vibrations

To further substantiate the unique vibrational features of *Varroa* gait and supplement the mite brood-comb data, recordings of mites walking on Petri-dish were used alongside two other species of invertebrates for a separate discrimination analysis.

Recordings of *Varroa* walking on Petri-dish were used from a previous study^44^, where individuals (collected from the bottom-board of a honeybee hive) were recorded walking on a Petri-dish with an attached accelerometer (see^44^ for full methods).

Carabid beetles (*n* = 10) and woodlice (*n* = 5) were collected using pit-fall traps placed in a wooded area of Nottingham Trent University Clifton campus. Each invertebrate was individually recorded walking on the same Petri-dish, with identical set-up, as used in Hall et al., (2021)^44^, amounting to 49 min of beetle recordings and 25 min of woodlouse recordings.

### Preparing the data for analysis

#### Mite, honeybee, and background

Mite, bee, and background categories of vibrations, identified and categorised both by critical listening and video evidence, were extracted from (i) accelerometer tracks of the two recordings where the mite and bee were in view, (ii) an additional recording where a mite was captured walking on brood-comb (which lacked bee related vibrations) and (iii) another where a bee could clearly be heard ‘scratching’ at the capping (no visual evidence of the bee emerging was collected, but this sample was known to contain bees only; no *Varroa* were present in the sample) (no. of bee category extracts = 242, no. of mite category extracts = 66, no. of background category extracts = 87). Each extract was 1 s in length, which was deemed to be a suitable duration for capturing multiple walking or bee emergence pulses in a single time window. The accelerometer data for each vibration was then transformed into a two-dimensional-Fourier-transform (2DFT)^63^, which best demonstrated the feature differences between each signal type (spectrogram images had previously been trialled separately and unsuccessfully).

Code written specifically for this study (Matlab 2020a), at Nottingham Trent University, was used for all analyses, and made available as indicated in the ‘data availability’ section. The horizontal axis of each 2DFT underwent interpolation to reduce the number of pixels that were unnecessarily present (over sampling) for analysis. Additionally, the 2DFTs were cropped to remove frequency bandwidths that contained no signal of interest (0–0.5 kHz and 4–24 kHz). All 2DFTs were then scaled by their maximum magnitude of acceleration to remove the signal strength from the discrimination exercise.

#### Mite, woodlouse, and beetle

For the invertebrate discrimination analysis, 3 recordings for each invertebrate were used to build a training database, consisting of 6 *Varroa* individuals, 3 woodlouse individuals and 3 beetle individuals. Periods of time where each animal was walking, uninterrupted (i.e., carrying out no other activity than locomotion, as beetles and woodlice were often seen antennating or scrabbling at the edge of the Petri-dish), were identified and extracted for use in the training database (TDB).

The analysis for each 2DFT that contributed to the TDB was carried out in the same way as for the mite, honeybee, and background discrimination. However, no interpolation of the data was necessary in this case. All 2DFTS were scaled by their mean magnitude of acceleration to remove signal strength from the discrimination exercise. Additionally, the averaged background noise was subtracted from each 2DFT to ensure that any differences in background vibration had no influence on the discrimination outcome, as the *Varroa* recordings were collected 2 years prior to those of the woodlouse and beetle.

### Signal analysis

#### Mite, honeybee, and background

The TDB which was comprised of mite, bee, and background 2DFTs, then underwent principal component (PCA) and discriminant function analysis (DFA)^43^ to determine which features in the database exhibited high variance, and reduce the size of the dataset using the features that demonstrated the highest variance for supervised classification. From this, a scatterplot was created to visualise each group of signals (mite, bee and background) in DF space. The two generic discrimination images, one for each dimension (vertical and horizontal discrimination between the data clusters) were also saved in 2DFT format for the next stage of signal analysis (see below). Optimum clustering of the groups and clarity of the discriminant 2DFTs were achieved using 25% of the total deviations found in the dataset.

The peripheral points of each of the individual signal clusters (found in the DF space scatterplot) were used to identify the boundaries of each group. The space within each cluster was then filled with a solid colour as in any data masking technique, to better emphasise the reach of the specific data within DF space. We here refer to each of these coloured areas as ‘masks’. Note that the periphery of the honeybee cluster was extended to include a larger portion of bee data, based on the outcome of projecting the full honeybee recordings, and critical listening to confirm the nature of the data. This extension to the mask provided a better representation of the DF space that honeybee vibrations inhabit (see Fig. 5 for the original clustering of the data, and Fig. 6 for the extended cluster based on the larger portion of data, which can be seen to fill the previously empty space between the honeybee cluster and the mite and background clusters).

New data points from the long-term brood-comb recordings (*Varroa* present and *Varroa* absent) could then be projected on this same DF space to determine if they fell into the correct cluster, to answer the question: can independent mite, bee, and background vibrations be correctly classified using this training data?

The same recordings that were used to create the TDB were also used for this analysis, but this time, exhaustively. As each recording lasted 30–120 min, they included many time durations of data that were known to contain mite, bee, or background vibrations, but had not been used to train the algorithm. Specific periods of accelerometer track that contained the vibrations of each type of signal were identified by critical listening and video visual evidence, and the same data preparation as that used for the training was undertaken, in 1-s-long Sects. (2DFT interpolation, normalisation, and cropping).

Separate cross-correlation product analysis was then implemented between the first 1-s-long 2DFT of the tested recording and the two discriminant 2DFT images acquired during PCA/DFA, to get the two DF space scores required for projection into DF space. This analysis was then repeated on the next 1-s-long extract of the recording, by moving on in time steps of 0.25 s. For each second of the recording, a set of co-ordinates were thus obtained, one for the vertical and one for the horizontal axis of the DF space scatterplot. This data-point could then be plotted to identify whether it had been correctly classified or not, based on its position in comparison to that of the scatterplot cluster masks. By searching recordings of vibration of a known category, correct discrimination could be established with high certainty, further supported through critical listening and visualisation of the data.

If a data-point fell outside of the cluster masks, or into the wrong mask, the TDB was then updated to include this particular data and the training run again. This gradually led to a more generic training, with clusters that covered a larger and more accurate area of DF space, enabling better discrimination. The two additional recordings that contained a mite walking in view and a bee scratching at the capping were eventually included in the TDB to obtain further improvement, as is typical of machine learning techniques. This iterative process can be repeated as long as the outcome of the training keeps providing scatterplots with negligible overlapping between different categories.

#### Mite, woodlouse, and beetle

For the invertebrate discrimination analysis, the same methods were implemented as for the mite, honeybee, and background discrimination exercise. However, as with the building of this TDB, during cross-correlation product analysis the tested 2DFT data was not interpolated, the averaged background was subtracted from each 2DFT, and each was scaled by the mean magnitude of acceleration.

The TDB originally consisted of 1 mite, 1 woodlouse, and 1 beetle recording, consisting of 3 mite individuals, 1 woodlouse individual, and 1 beetle individual. Following the testing of the data that contributed to the TDB, it was deemed appropriate to build upon it and add a further 3 recordings, one for each species (the updated TDB then contained the vibrations originating from 6 mite, 3 woodlice, and 3 beetle individuals). Following this, all data that was tested was either novel walking periods from recordings that contributed to the TDB, or walking periods taken from recordings that did not contribute to the TDB at all. The plotting outcome of these tested recordings can be seen in supplementary Table 1.

### Testing the TDB with novel brood-comb sample recordings

#### Mite, honeybee, and background

The improved TDB was then tested for its classification ability using additional long-term brood-comb sample recordings that did not contribute, at all, to its creation. Of the brood-comb samples that had been collected and recorded (discussed earlier in this methods section), eight that contained *Varroa* and eight that did not contain *Varroa* were tested in their entirety, to establish if a difference in the projected outcome could be found between mite “present” and “absent” samples.

A fourth category was also added to the TDB following this analysis, as one recording was found to contain particularly anomalous results. The full method of signal analysis was run once more using this four-category TDB, to determine whether an improved discrimination outcome could be achieved.

### Varroa gait comparison between brood-comb and Petri-dish

A comparison was made between *Varroa* gait on the irregular brood-comb substrate and a flat, homogenous surface (Petri-dish), to find out whether the walking behaviour seen on brood-comb is repeated on a more regular media.

Average spectral repetition features were established for ten 1-s-long 2DFTs per substrate. Additionally, the distribution of the time elapsed between each consecutive walking vibration pulse seen for these two 10 s walking extracts (brood-comb and Petri-dish) was quantitated, and the mode time established.

*Varroa* instantaneous velocity was also calculated for 10 s of walking behaviour on Petri-dish, by selecting a specific locus on the *Varroa* body (between the two antennae) and determining the coordinates of that pixel for every-other-frame of the 50 frames-per-second video. Average velocity was also quantitated using this information.

## Supplementary Information

Supplementary Video 1.

Supplementary Video 2.

Supplementary Information 1.

## Data Availability

All relevant data are in the paper and its supplementary material. Additional raw data in the form of accelerometer recordings that were used in the analysis for this study can be found on Figshare and all relevant Matlab code can be found on GitHub. Accelerometer recordings used to build the honeybee and *Varroa* training databases: 10.6084/m9.figshare.22761215, 10.6084/m9.figshare.22761269, 10.6084/m9.figshare.22761284, 10.6084/m9.figshare.22761320, 10.6084/m9.figshare.22761353. Accelerometer recordings used to build the invertebrate training databases: 10.6084/m9.figshare.22761461, 10.6084/m9.figshare.22761410, 10.6084/m9.figshare.22761473, 10.6084/m9.figshare.22761476, 10.6084/m9.figshare.22761482, 10.6084/m9.figshare.22761485, 10.6084/m9.figshare.22761488, 10.6084/m9.figshare.22761491. Accelerometer recordings for all brood-comb samples that contained *Varroa*: 10.6084/m9.figshare.22762139. Accelerometer recordings for all brood-comb samples that did not contain *Varroa*: 10.6084/m9.figshare.22762088. Accelerometer recordings for all invertebrates (beetle, woodlouse, *Varroa*) walking on Petri-dish that were used to test the success of the invertebrate TDB and machine learning algorithm: 10.6084/m9.figshare.22762214. All relevant code: https://github.com/HThomasntu/TDB_MLA_matlab_code.git.
