# Supplementary material for: Automated, non-invasive Varroa mite detection by vibrational measurements of gait combined with machine learning
Source: Sci Rep. 2023 Jun 23;13:10202. doi: 10.1038/s41598-023-36810-0 (PMC10290145; doi:10.1038/s41598-023-36810-0)
Supplement: Supplementary file 3 — Supplementary Information 1. [file 41598_2023_36810_MOESM3_ESM.docx]

**Supplementary Video 1:** A video to demonstrate the unique gait of a *Varroa*, captured as a 2DFT. The video first shows a bee emerging from her cell, followed by a *Varroa* walking and resting on brood-comb (panel ‘a’). These two successive videos run synchronously in time with panels ‘b’ and ‘c’, for the purpose of showcasing the vibrations created by the two animals visually, as they occur. Panel ‘b’ shows the accelerometer recording for each video, transformed into a 2DFT that updates in real time. The accelerometer data has here been cropped and filtered to remove redundant frequencies and background noise (high pass filter = 0 to 0.5 kHz, cropping = 4 to 24 kHz removed). Spectral repetition is shown on the x axis (Hz), and frequency is shown on the y axis (Hz). Panel ‘c’ showcases DF space. The centroid for each group (mite = black, bee = blue, background = cyan) is plotted to demonstrate where the clusters’ centre of gravity is in DF space. The red circle with black ‘tail’ directly represents the vibrations that occur in the video and follows their journey around DF space accordingly. This video demonstrates that the vibrations that we captured are unique to each animal and categorised correctly as such, i.e., when the bee produces vibrations, the red circle remains near the bee centroid. When the mite walks, the red circle moves towards the mite centroid, and when stationary, towards the background centroid.

**Supplementary Video 2:** A video to demonstrate the unique gait of a *Varroa*, captured as a 2DFT in comparison to that of a woodlouse and a beetle. This video has been created using the same analysis as Video 1. First, a *Varroa* is shown walking around the Petri-dish, followed by a woodlouse and then a beetle (panel ‘a’). As with supplementary Video 1, the successive videos run synchronously with panels ‘b’ and ‘c’, which show the accelerometer data (panel ‘b’) and the journey through DF space (panel ‘c’). The accelerometer data in panel ‘b’ has been cropped to remove background vibration that was interfering with the clarity of the 2DFT vibrational features between 0 to 260 Hz. Frequencies beyond 5600 Hz have also been removed, as there were no vibrational traces of interest beyond this. In panel ‘c’ the circles represent the centroid for each group, based on the DF scatterplot (see Figure 3) (mite = black, woodlouse = blue, beetle = cyan). This video demonstrates the differences in vibrational gait features for each animal, but also highlights some similarities between the woodlouse and beetle, as the red circle that represents the journey through DF space often moves between the two corresponding centroids. This is as expected based on the scatterplot, where the data from the two groups overlaps to some degree.

| 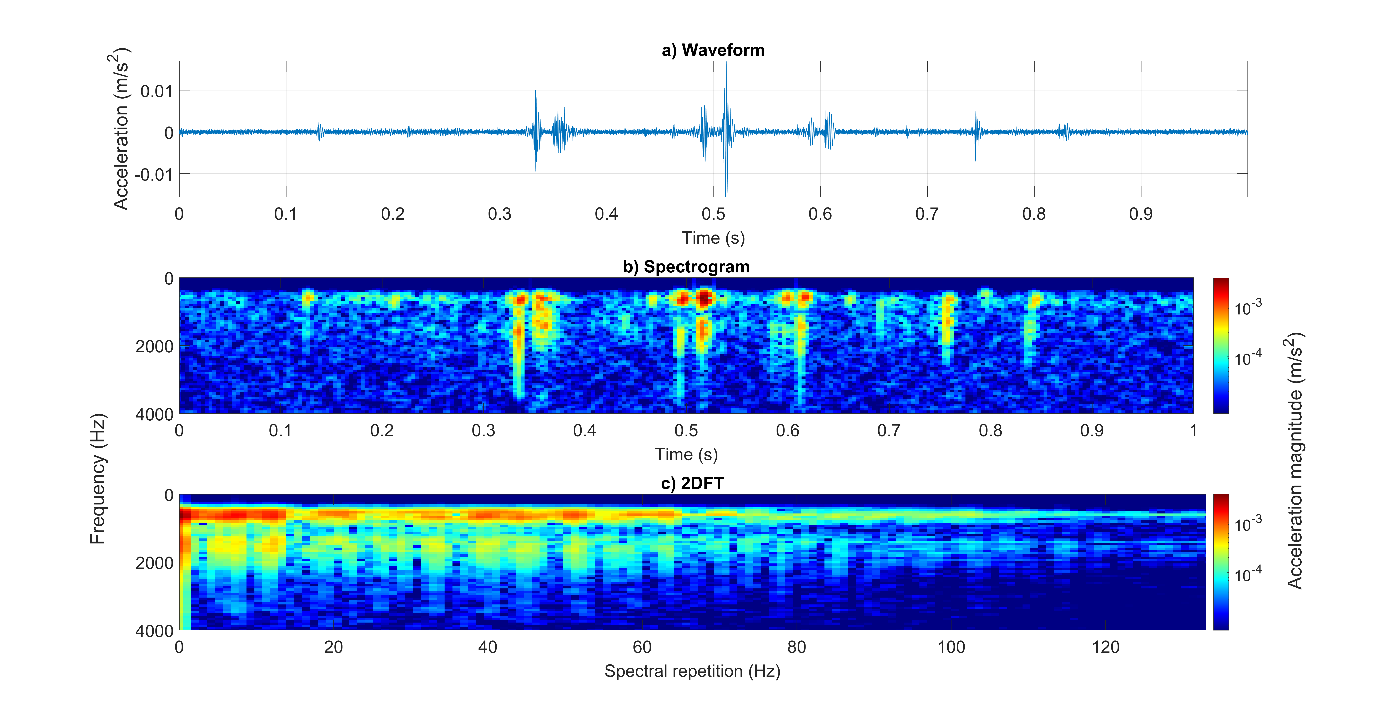 |
| --- |
| **Supplementary Figure 1:** A representative example of 1 second of honeybee emergence vibrations on brood-comb, viewed as an accelerometer waveform (panel ‘a’), spectrogram (panel ‘b’) and 2DFT (panel ‘c’). Magnitude of acceleration is in logarithmic (to the base 10), where dark red is the maximum (3.6 x 10^-3^ m/s^2^), and dark blue is the minimum (here forced to be 1/500 of the maximum). The spectrogram and 2DFT panels have been scaled identically and cropped to remove redundant frequencies (high pass filtered 0 to 500 Hz, cropped to remove frequencies above 4 kHz). |

| 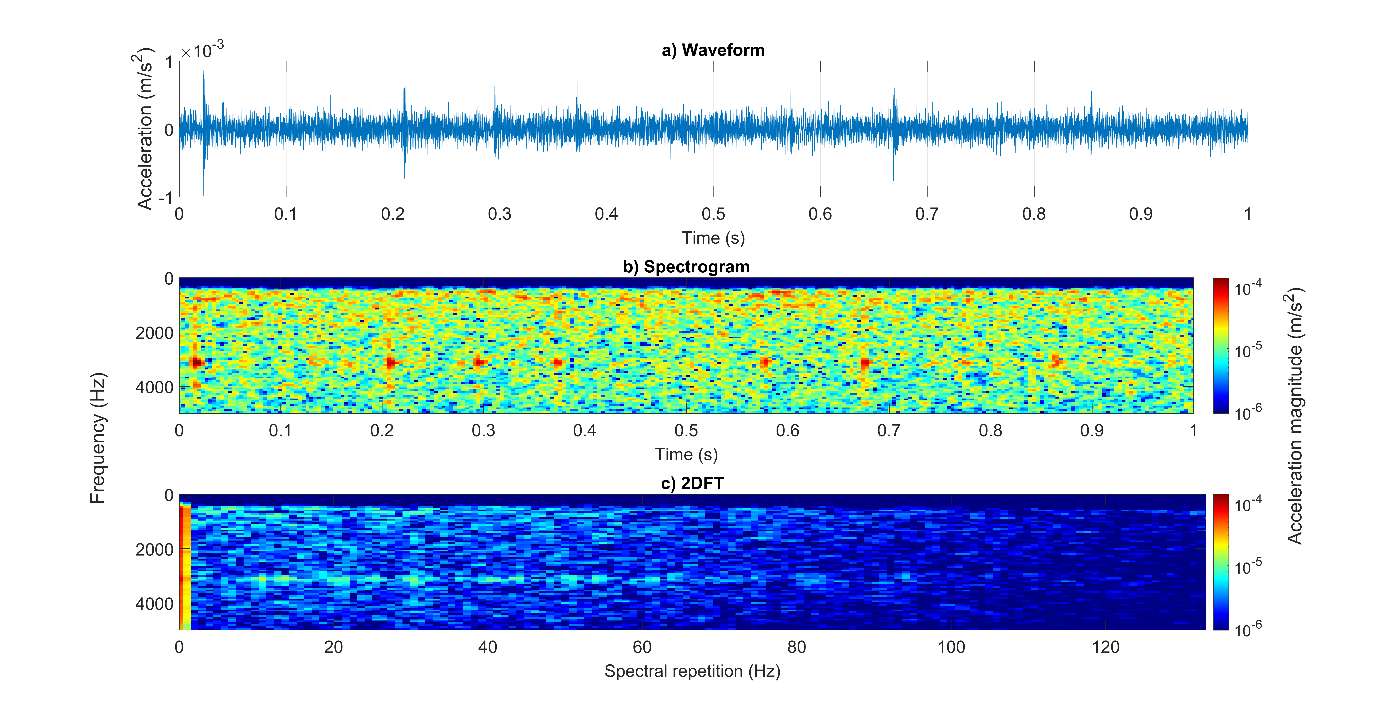 |
| --- |
| **Supplementary Figure 2:** A representative example of 1 second of *Varroa* mite walking vibrations on Petri-dish, viewed as an accelerometer waveform (panel ‘a’), spectrogram (panel ‘b’) and 2DFT (panel ‘c’). The data has been band-pass filtered (500 to 5000 Hz) to better showcase the vibrational pulses in the waveform panel. Magnitude of acceleration is in logarithmic (to the base 10), where dark red is the maximum (1.4 x 10^-4^ m/s^2^), and dark blue is the minimum (here forced to be 1/150 of the maximum). The spectrogram and 2DFT panels have been scaled identically for unbiased comparison. |

| 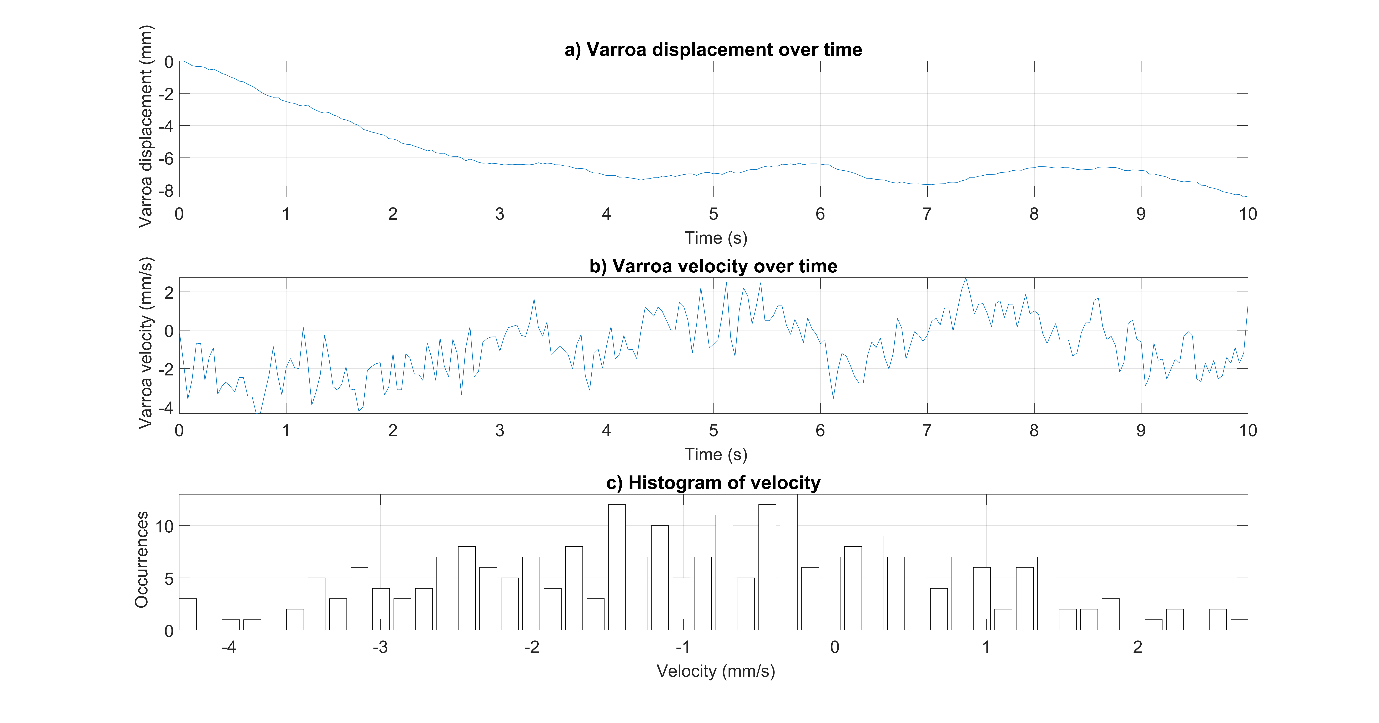 |
| --- |
| **Supplementary Figure 3:** The velocity of a *Varroa* mite walking on Petri-dish for 10 seconds. Panel ‘a’ demonstrates the displacement of the mite from A to B over time, as the mite did not walk in a straight line. Panel ‘b’ showcases the velocity of the mite during this period of movement. Panel ‘c’ highlights how often the mite moved at each speed, ranging from -4 to 2.6mm/s. |

| 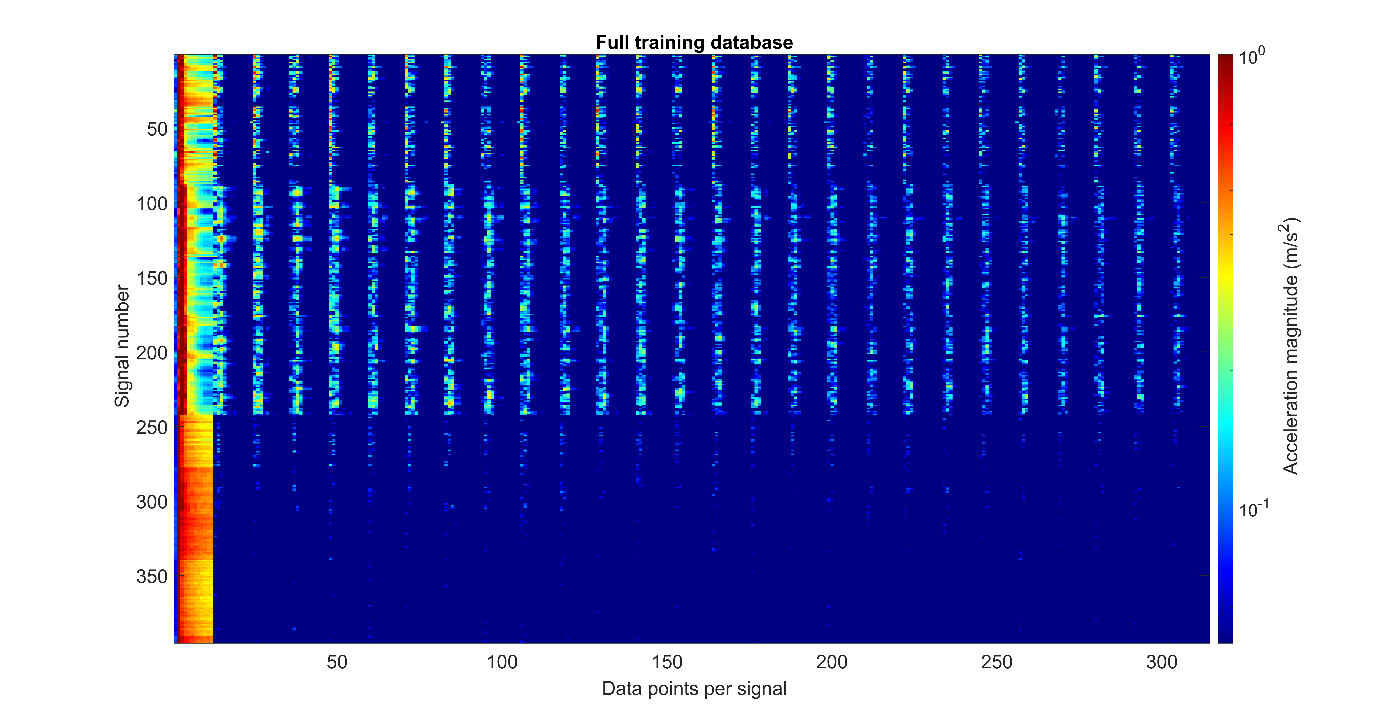 |
| --- |
| **Supplementary Figure 4:** All accelerometer data that contributed to the formation of the TDB. Each 1 second extract of honeybee, mite, and background signal can be seen here, with one signal per horizontal line of the figure (bee signals = 1 until 242, mite signals = 243 until 308, background signals = 309 until 395). Each extract has undergone 2DFT transformation, then been stretched into an array. They have then been interpolated and scaled identically, as well as filtered and cropped to remove redundant frequencies (high pass filter 0 to 0.5 kHz, cropping 4 to 24 kHz) for improved viewing of the training data. Magnitude of acceleration is normalised to its maximum, and logarithmic (to the base 10), with maximum magnitude seen as dark red (forced to be 1 m/s^2^) and minimum magnitude as dark blue (forced to be 1/20 of the maximum). |

| 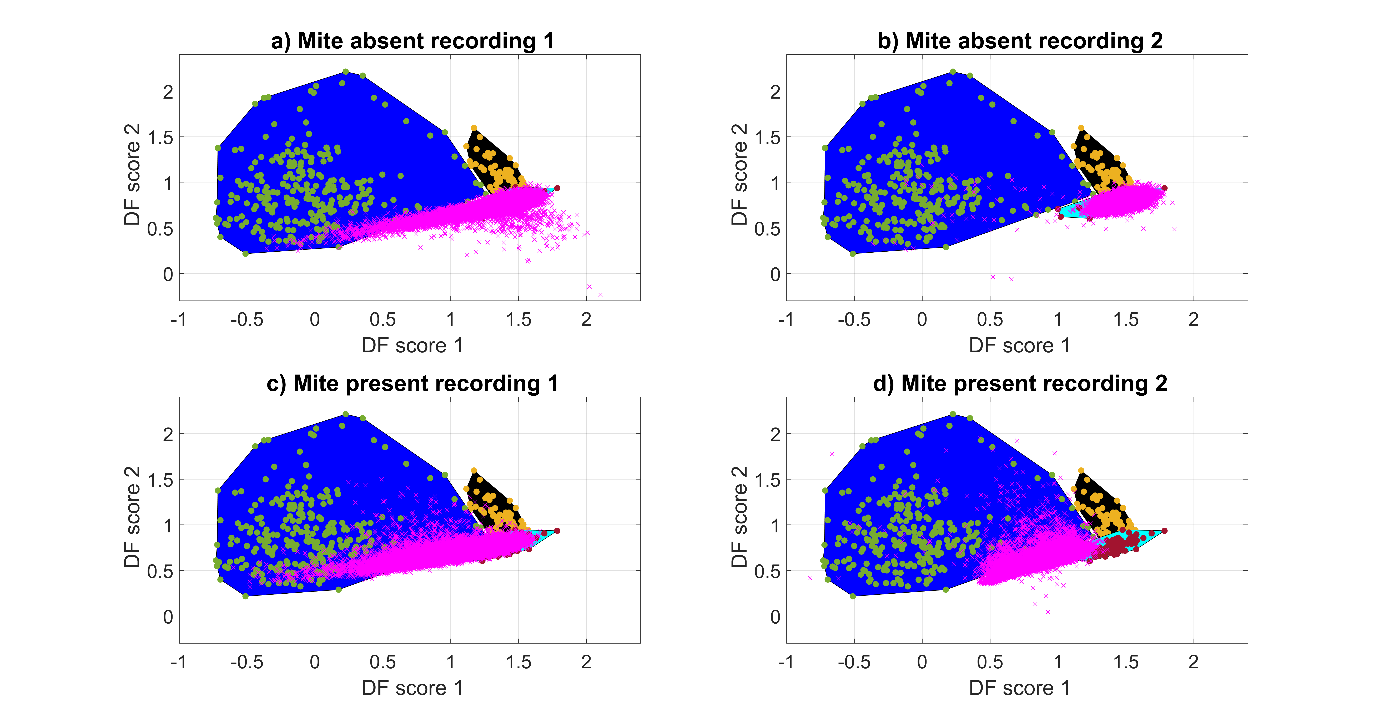 |
| --- |
| **Supplementary Figure 5:** The plotted classification outcome of two *Varroa* present (panels ‘a’ and ‘b’), and two *Varroa* absent (panels ‘c’ and ‘d’) recordings, chosen at random from the collection. The TDB scatterplot can be seen as: bee = blue, mite = black, background = cyan. The data from each long-term recording is projected onto the scatterplot as pink crosses. The lack of discriminatory features between the two types of recording can be seen here, particularly between panels ‘a’ and ‘c’. This figure also demonstrates the variation in datapoint spread (pink crosses) between the different recordings, likely (in part) resulting from the developing age of the bees within each sample, as larvae and pupae do not produce any vibrations (Ramsey, 2018 thesis). This may explain why panel ‘b’, for example, exhibits strong clustering of datapoints around the background mask. |

| 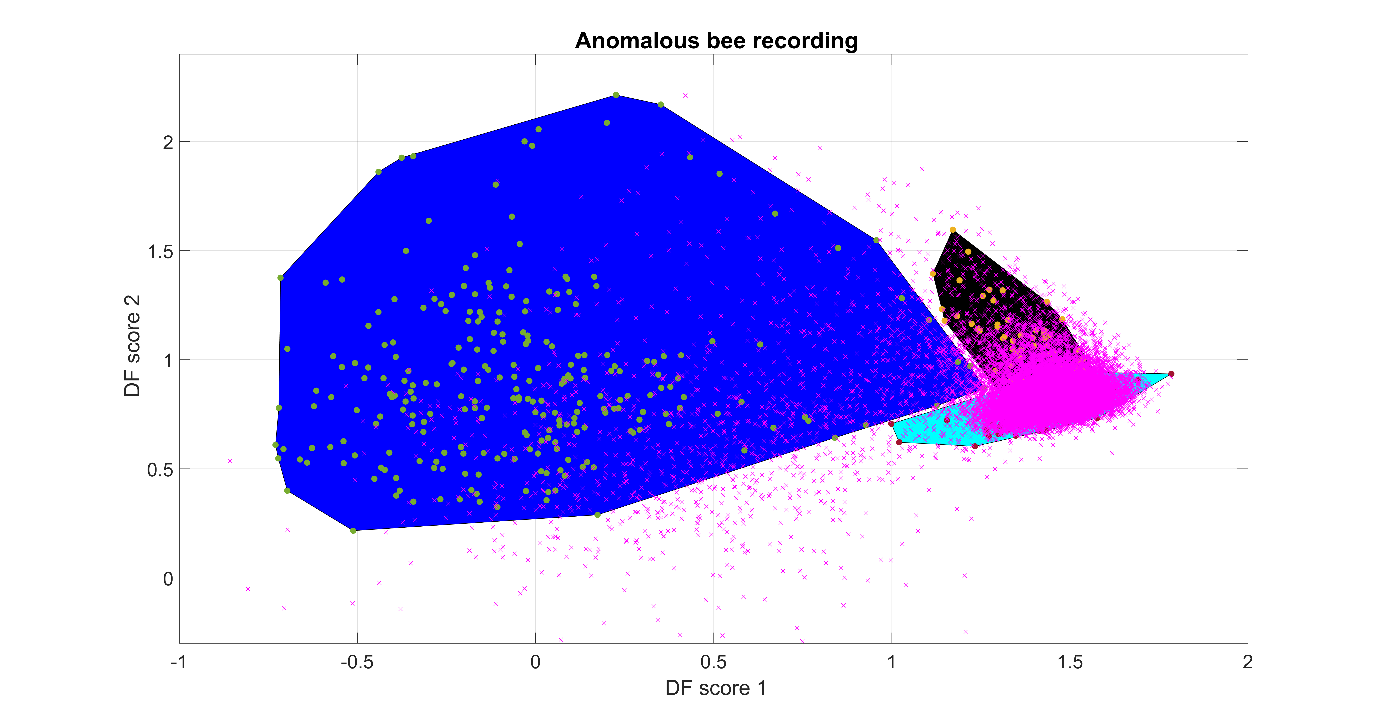 |
| --- |
| **Supplementary Figure 6:** The plotted classification outcome of the *Varroa* absent recording that presented anomalous clustering of points (pink crosses) over the mite mask (black). |

| 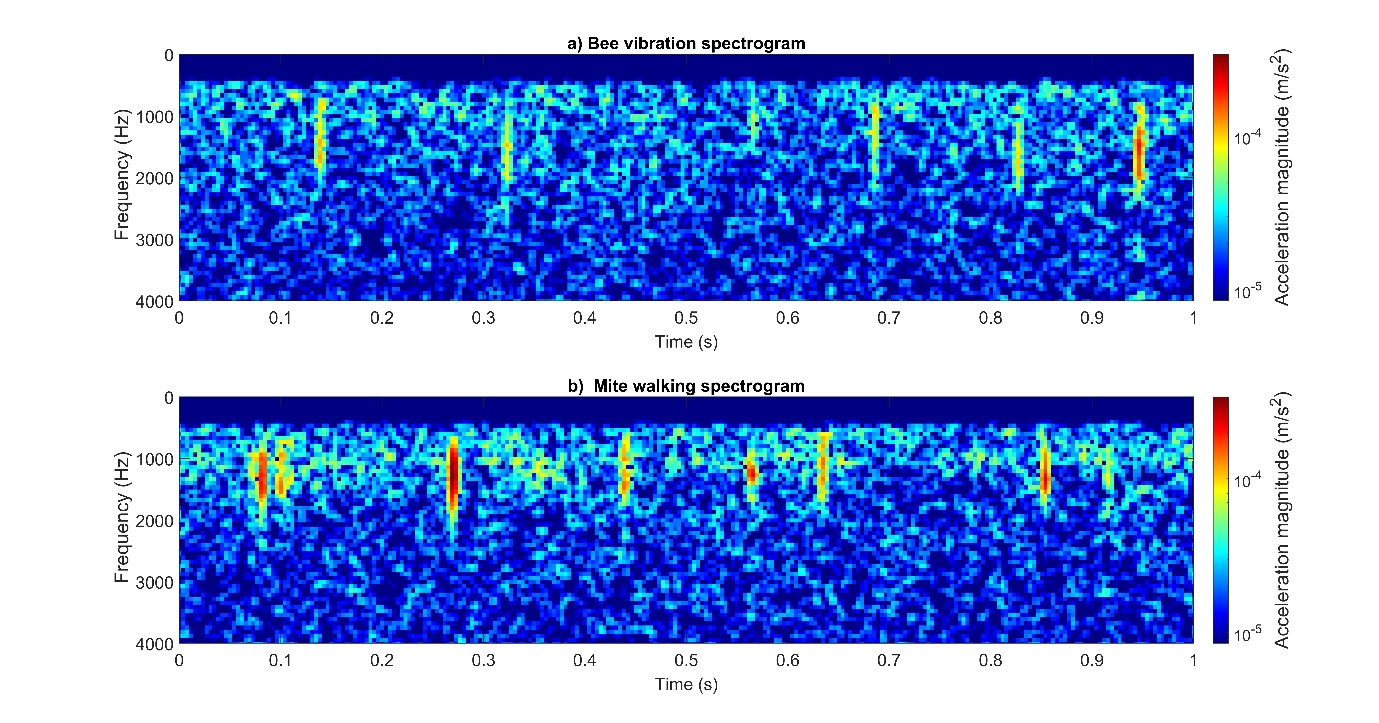 |
| --- |
| **Supplementary Figure 7:** Accelerometer data taken from the anomalous *Varroa* absent recording and a recording of a *Varroa* mite walking on brood-comb, viewed as spectrograms. Panel ‘a’ showcases a 1 second excerpt of bee vibrations and panel ‘b’ a 1 second excerpt of mite walking. Magnitude of acceleration is logarithmic (to the base 10) where maximum magnitude is dark red (3.4 x 10^-4^ m/s^2^) and minimum magnitude is dark blue (forced to be 1/40 of the maximum). Both panels are scaled identically for unbiased comparison. |

| 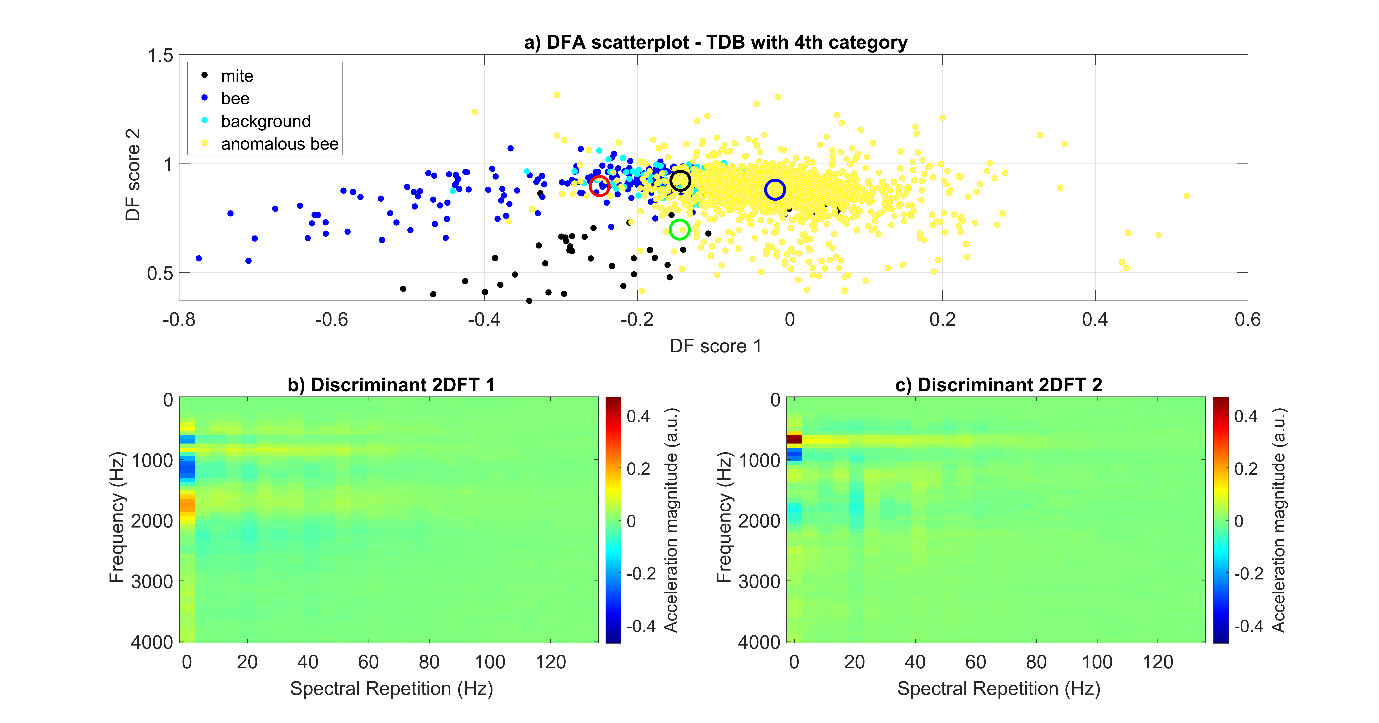 |
| --- |
| **Supplementary Figure 8:** A figure to show the results gathered from PCA/DFA of the 4 category TDB. Panel ‘a’ exhibits the scatterplot outcome of the 4 groups (bee = blue, mite = black, background = cyan, anomalous bee = yellow). The centroid for each data set is also shown for clarity of where the clusters fall in relation to one another (bee = red, mite = green, background = black, anomalous bee = blue). Panels ‘b’ and ‘c’ show the discriminant 2DFT images for the horizonal (‘b’) and vertical (‘c’) axis of DF space. Magnitude of acceleration is in arbitrary units, where dark red and dark blue indicate features of the 2DFT that have the strongest influence on the discrimination between the 4 groups of the TDB. Green is indicative of zero, i.e., those features that have no influence on the discrimination. The colour bar has been forced to show the maximum and minimum values as -0.47 to 0.47 a.u. for easier understanding of the colour coding. |

| Individual | % points in mite mask | % points in woodlouse mask | % points in beetle mask | % points falling in woodlouse/beetle overlapping area | % points falling outside of masks |
| --- | --- | --- | --- | --- | --- |
| Mite 1 (75s) | 82 | 2 | 0.3 | n/a | 15 |
| Mite 2 (27s) | 93 | 2 | 0 | n/a | 4 |
| Mite 3 (19s) | 100 | 0 | 0 | n/a | 0 |
| Mite 4 (17s) | 98 | 0 | 0 | n/a | 2 |
| Mite 5 (17s) | 49 | 9 | 0 | n/a | 42 |
| Mite 6 (28s) | 71 | 3 | 0 | n/a | 36 |
| Mite 7 (30s) | 90 | 0 | 0 | n/a | 10 |
| Mite 8 (33s) | 90 | 0.7 | 0 | n/a | 9 |
| Mite 9 (28s) | 20 | 23 | 0 | n/a | 56 |
| Mite 10 (10s) | 84 | 0 | 0 | n/a | 16 |
| Beetle 1 (16s) | 0 | 3 | 52 | 45 | 0 |
| Beetle 2 (16s) | 0 | 3 | 84 | 6 | 6 |
| Beetle 3 (69s) | 0 | 3 | 67 | 27 | 3 |
| Beetle 4 (19s) | 0 | 4 | 41 | 50 | 4 |
| Beetle 5 (61s) | 0 | 0 | 73 | 27 | 0 |
| Woodlouse 1 (27s) | 0 | 68 | 0.4 | 17 | 14 |
| Woodlouse 2 (72s) | 1 | 78 | 0.3 | 4 | 15 |
| Woodlouse 3 (70s) | 0 | 56 | 2 | 42 | 0.3 |
| Woodlouse 4 (70s) | 0 | 53 | 7 | 30 | 10 |

**Supplementary Table 1:** Percentage overlap of datapoints in each area of DF space for the recordings that contributed to the invertebrate gait TDB. Here, the results can be seen for the walking vibrations of 10 mites from 7 mite recordings, 5 beetles from 5 beetle recordings, and 4 woodlice from 4 woodlouse recordings. The time duration (seconds) of the tested walking data is in brackets next to the individual’s name. Fewer time periods could be tested for the beetle individuals as they regularly climbed at the Petri-dish wall in between short bursts of walking (1 to 2 seconds), and therefore prolonged periods of natural gait were less common. For the woodlouse and beetle walking data, some datapoints fell in an area of DF space where the two clustered areas overlapped. This was expected as the gait of the two species was audibly similar on some occasions.

| Recording | % points in mite mask | % points in bee mask | % points in background mask | % points falling outside of masks |
| --- | --- | --- | --- | --- |
| Honeybee 1 | 0.07 | 80 | 13 | 7 |
| Honeybee 2 | 0.01 | 62 | 15 | 23 |
| Mite 1 | 11 | 0.7 | 62 | 26 |
| Mite 2 | 12 | 5 | 61 | 22 |

**Supplementary Table 2:** Percentage overlap of datapoints into each area of the DF space scatterplot for each of the recordings that contributed to the mite, bee, and background TDB.

| Recording number (*Varroa* present) | % overlap *Varroa* mask |
| --- | --- |
| 1 | 0.4 |
| 2 | 1.3 |
| 3 | 0.3 |
| 4 | 0.04 |
| 5 | 0.7 |
| 6 | 0 |
| 7 | 0.02 |
| 8 | 0.07 |
| Recording number (*Varroa* absent) | **% overlap *Varroa* mask** |
| 1 | 0 |
| 2 | 0.04 |
| 3 | 0.04 |
| 4 | 0.07 |
| 5 | 10 |
| 6 | 0 |
| 7 | 0.03 |
| 8 | 0 |

**Supplementary Table 3:** Percentage overlap of datapoints into the mite mask for each of the 16 long-term recordings that did not contribute to the mite, bee, and background TDB.

| Recording number (*Varroa* present) | % overlap *Varroa* mask |
| --- | --- |
| 1 | 0 |
| 2 | 0.5 |
| 3 | 0.004 |
| 4 | 0 |
| 5 | 0.08 |
| 6 | 0 |
| 7 | 0 |
| 8 | 0.07 |
| Recording number (*Varroa* absent) | **% overlap *Varroa* mask** |
| 1 | 3 |
| 2 | 0 |
| 3 | 0 |
| 4 | 0 |
| 5 | 0.01 |
| 6 | 0 |
| 7 | 0 |

**Supplementary Table 4:** Percentage overlap of datapoints into the mite mask for each of the long-term recordings (8 *Varroa* present, 7 *Varroa* absent) that did not contribute to the mite, bee, and background TDB containing the fourth category.
